# Development of the larval anterior neurogenic domains of *Terebratalia transversa *(Brachiopoda) provides insights into the diversification of larval apical organs and the spiralian nervous system

**DOI:** 10.1186/2041-9139-3-3

**Published:** 2012-01-24

**Authors:** Scott Santagata, Carlee Resh, Andreas Hejnol, Mark Q Martindale, Yale J Passamaneck

**Affiliations:** 1 Long Island University-Post, 720 Northern Blvd., Brookville, NY 11709, USA; 2 Sars International Center for Marine Molecular Biology, University of Bergen, Thormøhlensgate 55, 5008 Bergen, Norway; 3 Kewalo Marine Laboratory, Pacific Biosciences Research Center, University of Hawaii, 41 Ahui Street, Honolulu, HI 96813, USA

**Keywords:** brachiopod, apical organ, nervous system, *Six3/6*, *NK2.1*, *orthopedia*, *fez*, *FoxG*

## Abstract

**Background:**

Larval features such as the apical organ, apical ciliary tuft, and ciliated bands often complicate the evaluation of hypotheses regarding the origin of the adult bilaterian nervous system. Understanding how neurogenic domains form within the bilaterian head and larval apical organ requires expression data from animals that exhibit aspects of both centralized and diffuse nervous systems at different life history stages. Here, we describe the expression of eight neural-related genes during the larval development of the brachiopod, *Terebratalia transversa*.

**Results:**

Radially symmetric gastrulae broadly express *Tt-Six3/6 *and *Tt-hbn *in the animal cap ectoderm. *Tt-NK2.1 *and *Tt-otp *are restricted to a central subset of these cells, and *Tt-fez *and *Tt-FoxQ2 *expression domains are already asymmetric at this stage. As gastrulation proceeds, the spatial expression of these genes is split between two anterior ectodermal domains, a more dorsal region comprised of *Tt-Six3/6, Tt-fez, Tt-FoxQ2*, and *Tt-otp *expression domains, and an anterior ventral domain demarcated by *Tt-hbn *and *Tt-NK2.1 *expression. More posteriorly, the latter domains are bordered by *Tt-FoxG *expression in the region of the transverse ciliated band. *Tt-synaptotagmin 1 *is expressed throughout the anterior neural ectoderm. All genes are expressed late into larval development. The basiepithelial larval nervous system includes three neurogenic domains comprised of the more dorsal apical organ and a ventral cell cluster in the apical lobe as well as a mid-ventral band of neurons in the mantle lobe. *Tt-otp *is the only gene expressed in numerous flask-shaped cells of the apical organ and in a subset of neurons in the mantle lobe.

**Conclusions:**

Our expression data for *Tt-Six3/6, Tt-FoxQ2*, and *Tt-otp *confirm some aspects of bilaterian-wide conservation of spatial partitioning within anterior neurogenic domains and also suggest a common origin for central *otp*-positive cell types within the larval apical organs of spiralians. However, the field of sensory neurons within the larval apical organ of *Terebratalia *is broader and composed of more cells relative to those of other spiralian larvae. These cellular differences are mirrored in the broader spatial and temporal expression patterns of *Tt-FoxQ2 *and *Tt-otp*. Corresponding differences in the expression of *Tt-hbn, Tt-NK2.1*, and *Tt-FoxG *are also observed relative to their respective domains within the cerebral ganglia of spiralians. Based on these data we argue that the anterior region of the bilaterian stem species included *Six3/6, NK2.1, otp, hbn, fez*, and *FoxQ2 *expression domains that were subsequently modified within larval and adult neural tissues of protostome and deuterostome animals.

## Background

Several hypotheses exist concerning the putative homology of various parts of larval and adult nervous systems found among bilaterian animals. Based on similar expression patterns of evolutionarily conserved transcription factors, some authors have concluded that the structure of the adult nervous system of the last common ancestor of the Bilateria included an anterior brain with three divisions and a distinct longitudinal ventral nerve cord [1-3], with bilaterians that exhibit a more diffusely organized central nervous system (for example, hemichordates) having acquired this characteristic secondarily [4]. Other hypotheses suggest that the last common ancestor of all bilaterians was more similar to extant acoelomorph flatworms that have an anterior compact brain with a centralized neuropil and parallel dorsal, ventral, and lateral longitudinal nerve cords [5,6], and that adult bilaterian 'brains' have evolved independently several times [7]. Although the phylogenetic position of the acoelomorph flatworms remains contentious [8], having a single anterior compact neuronal center may still be plesiomorphic for bilaterian animals. The origin of the anterior bilaterian nervous system may be an amalgamation of neural ectodermal domains that are positioned within the oral and aboral regions of a planula-like ancestor [9,10] that possessed only an intraepithelial nerve net. The majority of information on bilaterian neural development focuses on animals that form a centralized subepithelial nervous system and much less information is known about animals with diverse forms of intraepithelial nervous systems (so called 'skin brains' see [11]), whose significance in protostome evolution is rarely addressed.

Confounding issues regarding the origin of the adult bilaterian nervous system pertain to its spatial proximity and integration with components of a larval nervous system, particularly, the development of ciliated apical tuft cells, which numerous larval forms exhibit, and their relationship to the larval apical (sensory) organ where many neuronal cell bodies of larval forms are concentrated. Despite some shared developmental and structural features there is no uniform consensus regarding the homology of the larval aboral organs of cnidarian planulae with the larval apical organs of various bilaterians [12-15]. One obvious difference is that some paired-class homeobox genes involved with the development of larval apical organ and adult brain in bilaterians (such as *homeobrain, rx*, and *orthopedia*) are only expressed within oral ectoderm of cnidarians [16]. How the different ectodermal domains of a planula-like ancestor became coupled to the bilaterian anterior region remains an open question, but the resulting cellular domains within it are a combination of several different ciliary and neuronal cell types that may have been co-opted into unique apical structures several times (for example, see [17-19]). The neuronal compositions of bilaterian larval apical organs are clearly diverse, and the putative homology of various neurotransmitter-expressing cell types among evolutionarily distant larval types remains controversial. Furthermore, since similar morphologies among disparate larval forms may be the result of convergent evolutionary forces [20,21], testing these ideas requires finding novel methods and broad taxonomic sampling to evaluate the homology of these intriguing larval structures.

The larval apical organs of phoronids and brachiopods are relevant to the evolutionary reconstruction of bilaterian brains. Although the evolutionary relationships within phoronids and brachiopods [22,23] as well as their exact sister group position are still under debate [24-26], phoronids and brachiopods clearly reside within the assemblage of protostome animals known as the Lophotrochozoa or Spiralia. In light if this, developmental and structural traits (cleavage patterns, mesoderm formation, morphology of the coelomic cavities, and ciliated bands comprised of monociliated cells) that once aligned phoronids and brachiopods with deuterostomes have been largely disproven or interpreted as the result of convergent evolution [27-30]. The presence of numerous (thirty or more) serotonergic cells in the larval apical organs of phoronids (previously referred to as the apical ganglion, but usage of this term has been criticized, see [31]) has also been interpreted as a deuterostome-like trait [32,33]. However, further investigation showed that the types of serotonergic cells within the actinotroch apical organ correspond more to serotonergic cell types within the apical organs of annelids and mollusks [34,35]. The apical organs of phoronid larvae also differ from those of echinoderm and hemichordate larvae in that apical organs of actinotrochs are comprised of a tombstone or U-shaped field of neuronal cells that send processes into a central neuropil [34,36], and these neuronal cell bodies do not originate within ciliated bands. Similar structural features are found in the larval apical organs of brachiopods, although neurotransmitter expression within apical neuronal cell types varies among systematic groups [37-39].

Considering all of these structural and biochemical differences among bilaterian larval apical organs, evaluating the homology of cell types and the complex neuronal centers they make up becomes problematic as there are no universally agreed upon criteria for discriminating homologous neuronal cell types (but see [40]). Some studies have tried to make a connection between the expression of select patterning genes and the specification of neuronal cells with conserved neurotransmitter expression types (for example, serotonin or vasotocin, see [41,42]), however the complete gene regulatory networks that specify the great majority of anterior larval neurosecretory cell types remain unknown. What has been shown more recently is the broad conservation of genes involved in the specification of both larval and adult anterior neural ectoderm, such as *Six3/6, homeobrain*, and *NK2.1 *[3,43-45]. Even if a direct connection between the expression of neural ectodermal-related genes and the neural architecture of various larval apical organs remains elusive, one plausible hypothesis is that evolutionary modifications to the combinatorial expression domains of these genes have contributed to cellular diversity of larval apical organs.

Evaluating alternative viewpoints regarding either the wide-scale homology or independent origin of larval apical organs requires more developmental data focused on the molecular specification of various neurogenic tissue domains from additional bilaterian animals with structurally diverse larval nervous systems. Recent reports on the development and structure of the sensory cells and larval nervous system in brachiopods [39,46] suggests that the broad specification of the neural ectoderm, simple ciliary photoreceptors, and wide-spread usage of conserved neurotransmitters within their basiepithelial nervous systems may yield key insights into the evolution of larval traits. Although the larval nervous systems of brachiopods have distinct features from what is observed in the larval forms of both spiralians and deuterostomes, all of these larval types share some morphologically similar peptidergic neuronal cell types [35,39]. How these cell types are deployed within anterior neural tissues may yield key insights into the origin and diversification of bilaterian larval nervous systems. The putative homology of neural structures within the larval nervous systems of brachiopods and spiralians (for example, apical organ, cerebral ganglion, and ventral nerve cord) is also not understood. Furthermore, because the spatial expression of particular neural-related genes (for example, *NK2.1*; [41]) differs in ambulacralian deuterostomes and spiralians (gastropods and polychaetes), the larval apical organs of protostomes and deuterostomes are generally considered not to be homologous structures (but see [13]). However, this conclusion does not fully take into account the different neural ectodermal domains that comprise adult, bilaterian anterior nervous systems, and how they are sometimes integrated with or separate from the larval nervous system. For these reasons we have investigated the development of the larval nervous system in the rhynchonelliform brachiopod, *Terebratalia transversa*, by analyzing the expression patterns of eight genes known to have roles in specification and differentiation of anterior neural tissues in other bilaterian animals.

## Results

### Gene alignments and trees

Full length or partial cDNA sequences for *Terebratalia Erebratalia transversa *orthologs of the transcription factors *forebrain zinc-finger *(*fez*), *Forkhead G *(*FoxG*), *Forkhead Q2 *(*FoxQ2*), *homeobrain *(*hbn*), *NK2.1, orthopedia *(*otp*), and *Six3*/*6*, and the synaptic vesicle-localized transmembrane protein *synaptotagmin 1*, were isolated by rapid amplification of cDNA ends (RACE). Orthology of each gene to representatives from other metazoan taxa was confirmed by Bayesian analysis of phylogenetics (Additional files 1, 2, 3, 4, 5, 6). *T. transversa *orthologs are subsequently referred to as *Tt*-*fez, Tt*-*FoxG, Tt*-*FoxQ2, Tt*-*hbn, Tt*-*NK2.1, Tt*-*otp, Tt*-*Six3*/*6*, and *Tt*-*synaptotagmin 1*.

### General aspects of gastrulation and larval development

**Figure 1:**
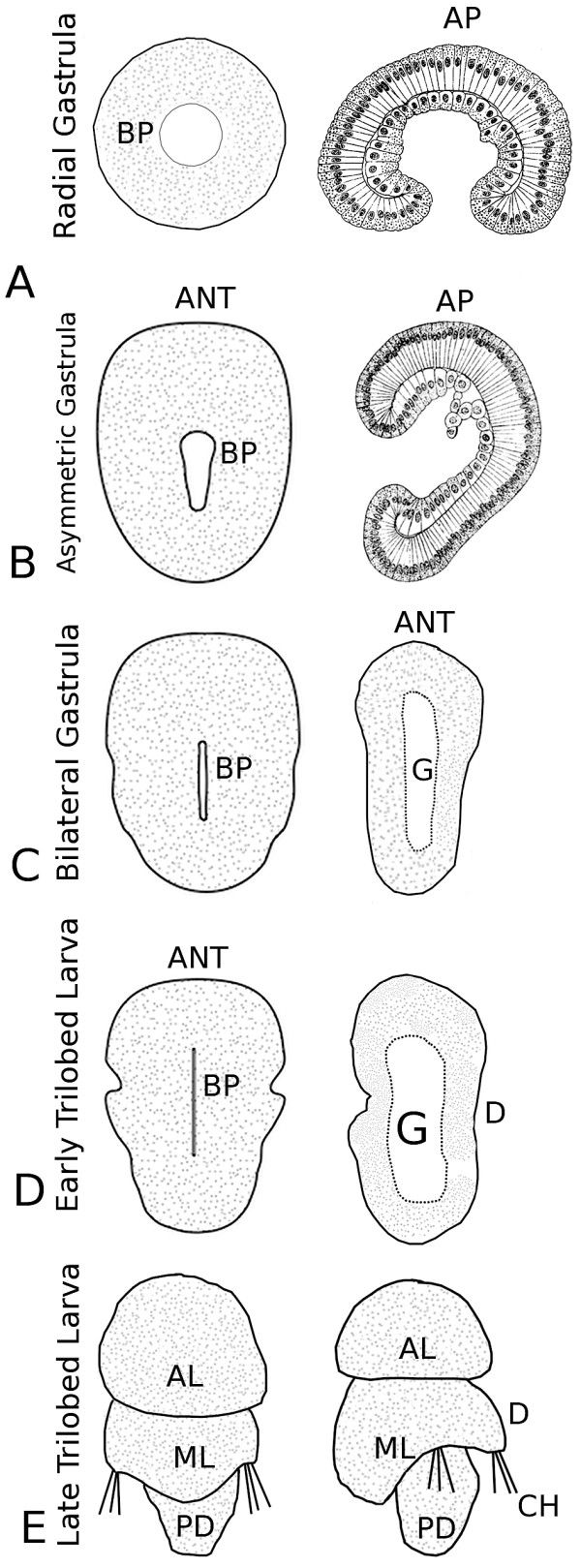
**General aspects of gastrulation and larval development of *Terebratalia transversa***. Each panel consists of a blastoporal (left) and a corresponding lateral (right) view of a particular developmental stage. **(A) **Radial gastrula stage, the animal pole (AP) is at the top. **(B) **Asymmetric gastrula stage showing the shift of the animal pole toward the presumptive anterior of the embryo (ANT). **(C) **Bilateral gastrula stage, when the blastopore (BP) is a narrow slit. **(D) **Early trilobed larval stage that begins to demarcate the apical, mantle, and pedicle lobes (AL, ML, and PD, respectively) of the larva. The gut (G) is a blind-ended sac. **(E) **The late trilobed larval stage has a larger ventral mantle lobe relative to the dorsal side (D) that also bears chaetae (CH).

General aspects of early development and the origin of embryological tissues have been described [47], and so only some aspects of gastrulation and larval development will be covered here. Once the embryo has reached a hollow blastula stage consisting of a single epithelial layer, gastrulation begins with the embolic invagination of the vegetal side of the blastula until the presumptive mesodermal and endodermal tissues take up much of the blastocoelic space (radial early gastrula stage; See Figure 1A). At this stage the tip of the archenteron makes contact with the animal pole of the embryo and the shape of the early gastrula is still radially symmetrical. As gastrulation continues, the archenteron bends toward the presumptive anterior end of the embryo. As the embryo elongates along the presumptive anterior-posterior axis (asymmetric middle gastrula; Figure 1B, C), the blastopore extends into a narrow oval (BP, Figure 1B, C). Later in gastrulation, the dorsal side of the embryo flattens and the more ventral tissues near the site of the blastopore move toward the midline and curve inwardly. At this stage the blastopore is progressively narrowed into a slit-like opening (bilateral late gastrula stage; Figure 1C). As the shape of the late gastrula acquires the three body regions (apical, mantle, and pedicle lobes) typical of the early trilobed larva, the slit-like blastopore is progressively closed from posterior to anterior leaving only a small circular oral opening in the apical lobe leading into the blind-ended gut (early trilobed larval stage; Figure 1D). During larval development the most anterior portion of the apical lobe differentiates into a rounded dome that sits on the wider cylindrically shaped portion that will include the anterior transverse ciliated band. The mantle lobe extends posteriorly to partially cover the posterior pedicle lobe and develops paired dorsal and medial chaetal sacs. The pedicle lobe narrows at its posterior end and divides into muscular and glandular portions near the time of metamorphic competence (late trilobed larval stage; Figure 1E).

**Figure 2:**
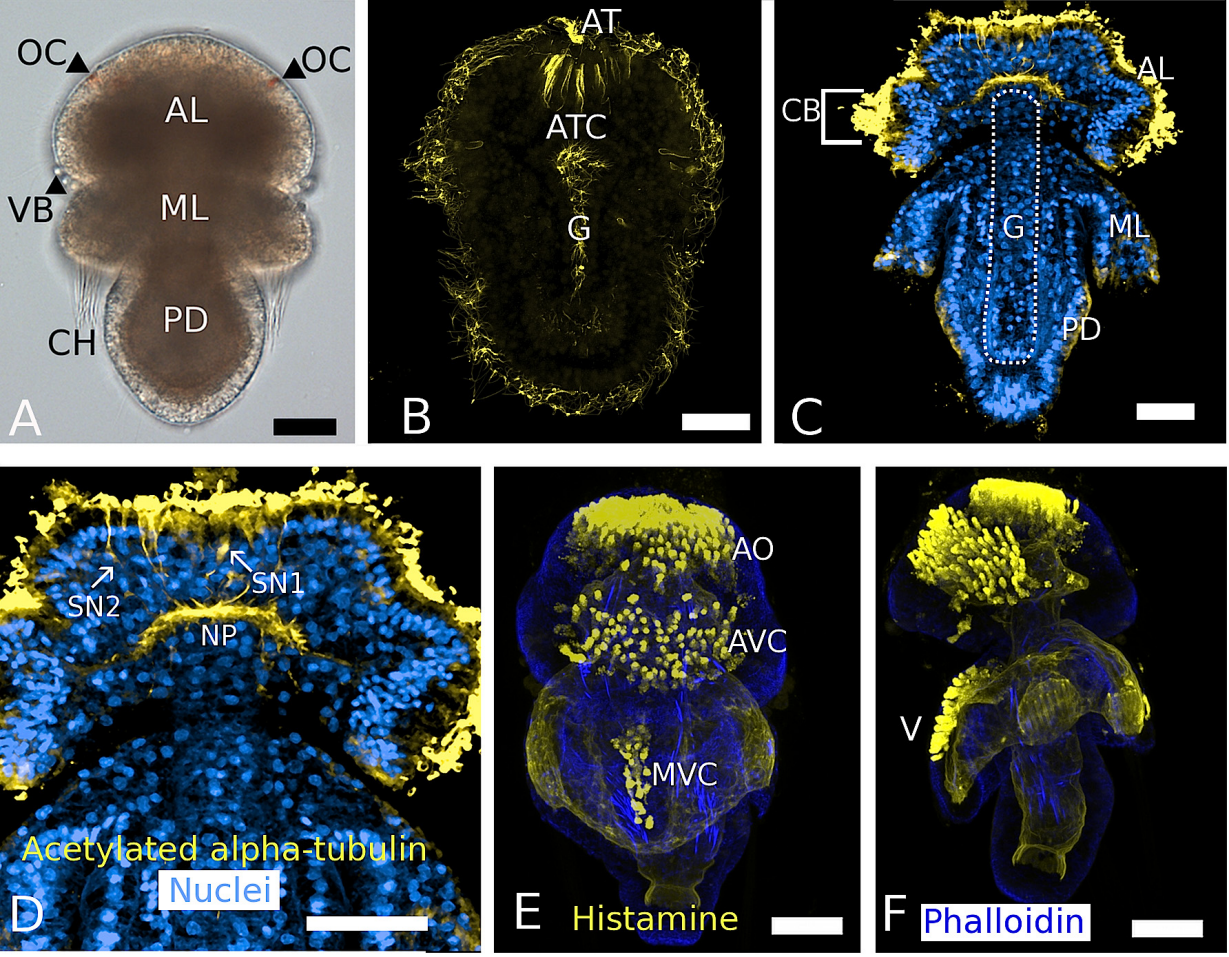
**Cytological and anatomical aspects of the late gastrula and trilobed larva of *Terebratalia transversa***. **(A) **Light micrograph of a late trilobed larva with ocelli (OC) on the dorsal side of the apical lobe (AL). Vesicular bodies (VB) and other epidermal cells line the border between the apical and mantle lobe (ML). The mantle lobe has four chaetal sacs with long chaetae (CH). The posterior pedicle lobe (PD) will attach the larva to the substrate at metamorphosis. **(B) **Ventral view of a bilateral late gastrula stage labeled for acetylated α-tubulin with a long ciliary tuft (AT) produced by specialized cells with recessed ciliary rootlets (ATC). **(C, D) **Partial frontal z-projections depicting aspects of late larval anatomy such as the cilia of the anterior transverse ciliated band (CB) and sensory neurons (SN1 and SN2) within the apical organ (AO) that send axonal fibers into the central anterior neuropil (NP). The larva is nonfeeding, but does develop a blind-ended gut (G). **(E, F) **Complete z-projections of the histaminergic nervous system of the late trilobed larva. Cell borders and some larval muscles are stained with phalloidin. There are at least 70 histaminergic cells in the apical organ (AO), approximately 60 histaminergic cells in the broad ventral region of the apical lobe (AVC), and also approximately 30 histaminergic cells (MVC) in a mid-ventral region (V) in the mantle lobe. All scale bars = 25 μm.

Other general features of late stage *Terebratalia transversa *larvae are the pigmented ocelli that are found on the dorsal side of the apical lobe (OC, Figure 2A) and a ring of vesicular bodies that border the posterior edge of the apical lobe where it meets the mantle lobe (VB, Figure 2A). During the development of the larval apical organ, a central group of cells within it produces a long ciliary tuft (AT, Figure 2B) and surrounding it are at least ten cells with recessed ciliary rootlets (ATC, Figure 2B) that label distinctively for acetylated α-tubulin. The long ciliary tuft centrally located in the apical organ is present in later trilobed larval stages [48], but the morphological complexity of apical cell types changes in later trilobed larval stages as the apical organ broadens into its final state. The apical lobes of late trilobed larval stages are generally monociliated, but also have a band of longer cilia in a transverse row in the posterior portion of the apical lobe (CB, Figure 2C). This larval type is nonfeeding, but does retain the blind-ended larval gut (G, Figure 2C) and extensive musculature especially in the mantle and pedicle lobes that are mainly involved in morphogenetic movements at metamorphosis [39]. Late trilobed larvae have a broad apical organ that contains numerous (at least thirty) monociliated sensory neurons with at least two morphological types that send axonal fibers into a central neuropil [39]. Only a subset of these neurons within the apical organ is shown in Figure 2D (SN1 and SN2). Surrounding these sensory neurons are other cell types that contribute to the pseudostratified epithelium anterior to the central neuropil (Figure 2D). Many (if not all) of the cells that surround the acetylated α-tubulin-labeled neurons are histaminergic in the broad apical organ (AO, Figure 2E). In late trilobed larval stages, the nervous system has at least three distinct basiepithelial neural domains, two of which are anterior (dorsal and ventral), and one that is mid-ventrally located on the mantle lobe of the larval body (Figure 2E, F). Additional details of the histaminergic nervous system of the competent larva are described in Santagata [39], but some features important for the interpretation of the gene expression patterns described here are the more dorsal position of the apical organ composed of at least seventy histaminergic cells (AO, Figure 2E, F), the wide histaminergic cell cluster on the ventral side of the apical lobe (approximately sixty cells, AVC, Figure 2E, F), and the histaminergic cell cluster on the ventral midline of the mantle lobe (thirty cells, MVC, Figure 2E, F).

### Gene expression patterns during gastrulation and larval development as detected by whole mount *in situ *hybridization

#### Tt-homeobrain

**Figure 3:**
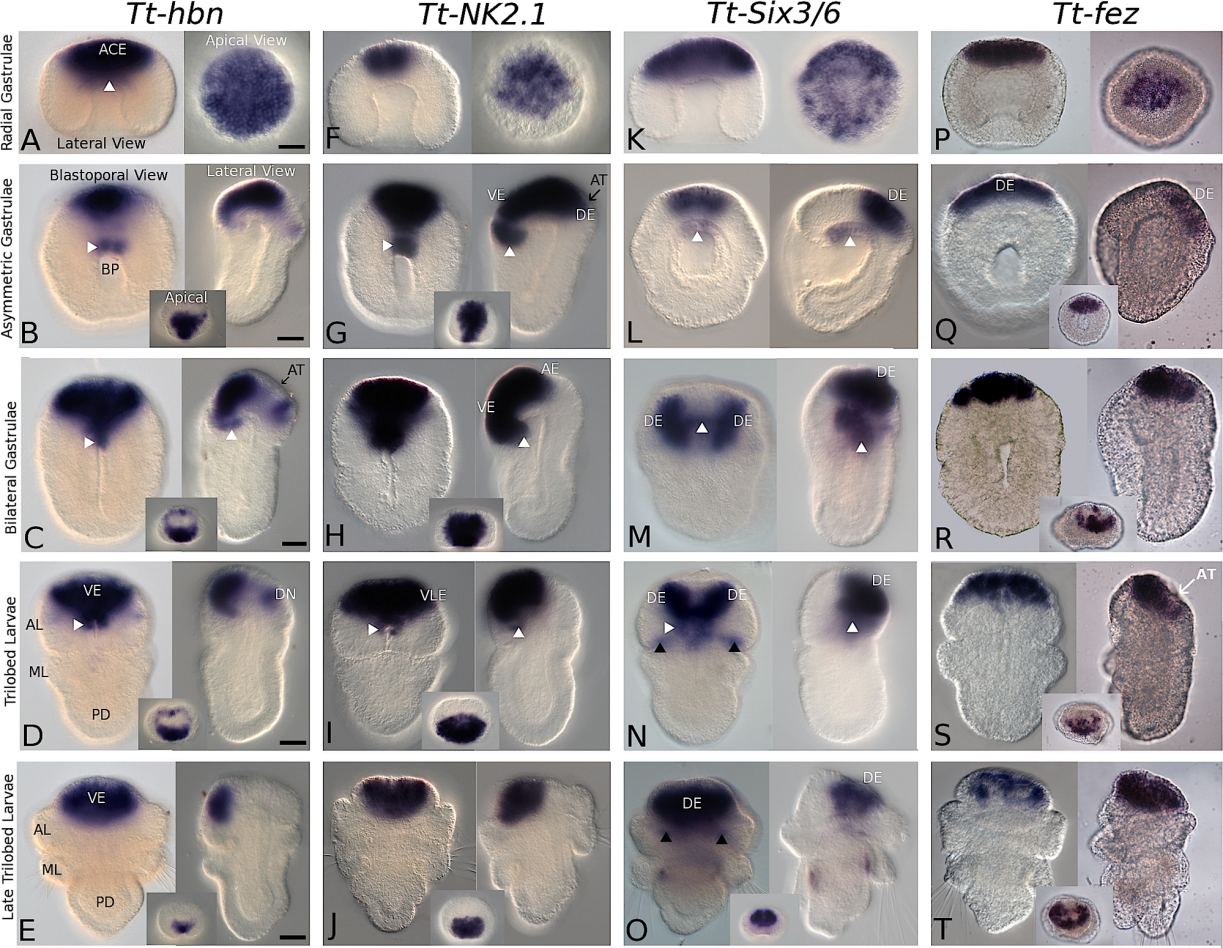
**Expression patterns of *Tt*-*hbn, Tt*-*NK2.1, Tt*-*Six3/6*, and *Tt*-*fez *in the embryos and larvae of *Terebratalia transversa***. Abbreviations: ACE, animal cap ectoderm; AL, apical lobe; AT, apical tuft; BP, blastopore; DE, anterior dorsal ectoderm; DN, anterior dorsal ring of ectoderm; ML, mantle lobe; PD, pedicle lobe; VE, anterior ventral ectoderm; VLE, anterior ventrolateral ectoderm. All scale bars = 25 μm.

In early radial gastrula stages *Tt*-*hbn *is expressed in the roof of the archenteron (arrow, Figure 3A) and broadly throughout in the animal cap ectoderm (ACE, Figure 3A), which will form the presumptive anterior ectoderm of the larva. This broad zone of expression is largely retained in subsequent stages of gastrulation, then shifting to the ventrolateral ectoderm. Two additional domains of expression appear in the asymmetric middle gastrula stage, one at the anterior lip of the blastopore and one in the dorsal ectoderm (Figure 3B). By the bilateral late gastrula stage the blastoporal expression coalesces with the ventral ectodermal domain, while the dorsal domain expands laterally to connect with the broad ventral and anterior domains, circumscribing the dorsal anterior region, from which expression is absent (Figure 3C). The broad ventral ectodermal domain of expression (VE, Figure 3D), and the dorsal ectodermal ring connecting to it (DN, Figure 3D), persists through the early trilobed larval stage. In late larval stages, expression of *Tt-hbn *is localized to only the ventrolateral ectoderm of the apical lobe (Figure 3E).

#### Tt-NK2.1

At the radial gastrula stage expression of *Tt-NK2.1 *is restricted to a slightly asymmetric, central region of the animal cap ectoderm (Figure 3F). Expression of *Tt-NK2.1 *dramatically broadens in later developmental stages and becomes more similar to that of *Tt-hbn. Tt-NK2.1 *is expressed broadly in asymmetric middle stage gastrulae within the anterior region of both the ventral and dorsal ectoderm (VE and DE, Figure 3G), including the region of the apical tuft, as well as endodermal tissues in the anterior lip of the blastopore (arrow, Figure 3G). Expression of *Tt-NK2.1 *shifts ventrally in bilateral late stage gastrulae, but is still broadly maintained in both the ventral and anterior ectoderm (Figure 3H). Trilobed larval stages express *Tt-NK2.1 *mainly in the anterior ventral ectoderm reminiscent of *Tt-hbn *expression, but in contrast to *Tt-hbn, Tt-NK2.1 *expression is also found more broadly in the anterior ventrolateral ectoderm (VLE, Figure 3I). Late trilobed larval stages express *Tt-NK2.1 *in the same anterior ventrolateral portions of the apical lobe including both surface and deeper epithelial tissues (Figure 3J).

#### Tt-Six3/6

*Tt*-*Six3/6 *is broadly expressed in the animal cap ectoderm of the radial early gastrula, similar to but more broadly than that of *Tt-hbn *(Figure 3K). Ectodermal expression shifts anteriorly to only the dorsal portion of the anterior ectoderm in the asymmetric middle stage gastrula (DE, Figure 3L). An additional domain in the underlying anterior endoderm is also present (white arrow, Figure 3L). The ectodermal expression of *Tt-Six3/6 *in the bilateral late stage gastrula splits into two bilaterally symmetrical masses flanking the midline (DE, Figure 3M). These dorsolateral domains of ectodermal expression in the anterior of the apical lobe, and the underlying endodermal domain persist through larval development. The medial gap in expression between the two dorsolateral ectodermal domains decreases as larval development continues (Figure 3N) until eventually the dorsomedial portion of the apical lobe also expresses *Tt-Six3*/*6 *(DE, Figure 3O). The expression of *Tt-Six3/6 *includes the anterior-most surface epithelial layer as well as subepithelial cell layers that do not extend to either the dorsal or ventral epithelial surface of the apical lobe. Early and late trilobed larval stages also have an additional ectodermal domain of *Tt*-*Six3/6 *expression in the posterior portion of the apical lobe, close to the junction with the mantle lobe (black arrow, Figure 3N, O).

#### Tt-fez

*Tt-fez *is expressed in a triangular-shaped central region of the animal cap ectoderm (Figure 3P). In the asymmetric middle stage gastrula, *Tt-fez *expression is limited to the anterior dorsal ectoderm (DE, Figure 3Q). Expression of *Tt*-*fez *in this region is maintained in the bilateral late stage gastrula and early trilobed larval stages, but the lateral edges of the expression domain bend dorsally into a 'U' shape (Figure 3R, S). In the late trilobed larval stages, *Tt-fez *is expressed in the lateral edges of the dorsal ectoderm creating a nearly complete ring-shape (see apical view, Figure 3T). The central region of the dorsal neural ectoderm, near the region of the apical tuft, does not express *Tt-fez*.

#### Tt-FoxQ2

**Figure 4:**
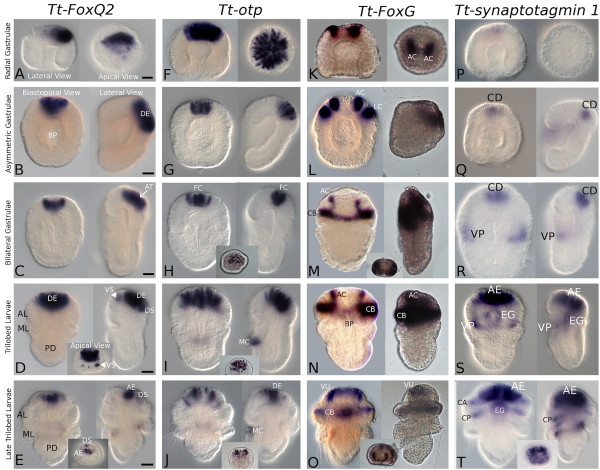
**Expression patterns of *Tt*-*FoxQ2, Tt*-*otp, Tt*-*FoxG*, and *Tt*-*synaptotagmin 1 *in the embryos and larvae of *Terebratalia transversa***. Abbreviations: AC, spots of animal cap ectoderm; AE, anterior ectoderm; AL, apical lobe; AT, apical tuft; BP, blastopore; CA, anterior zone within the transverse ciliated band; CB transverse ciliated band region; CD, central anterior ectoderm; CP, posterior zone within the transverse ciliated band; DE, anterior dorsal ectoderm; DS, anterior dorsal spots of ectoderm; EG, ectodermal cells around the anterior tip of the foregut; FC, flask cells; LC, ventrolateral expression spots; MC, expression spots on the mid-ventral region of the mantle lobe; ML, mantle lobe; PD, pedicle lobe; VP, ventral posterior ectoderm in the apical lobe; VU, anterior ventral U-shaped region; VS, anterior ventral expression spots. All scale bars = 25 μm.

Radial early stage gastrulae express *Tt-FoxQ2 *in an asymmetric domain shifted toward the presumptive dorsal end of the anterior ectoderm more so than what is observed for *Tt-NK2.1 *(Figure 4A). Asymmetric middle stage gastrulae express *Tt-FoxQ2 *in a subset of anterior dorsal ectodermal domain (DE, Figure 4B) similar to the dorsal ectodermal domains of *Tt-Six3/6 *and *Tt-fez *(Figures 3L, Q). This expression pattern is more constricted in bilateral late stage gastrulae as it is found in a more central region of the dorsal ectoderm that includes the region of the apical tuft (AT, Figure 4C). Early trilobed larvae express *Tt-FoxQ2 *more broadly in the anterior dorsal ectoderm of the apical lobe of the larva as well as a few small dorsal and ventral spots of expression (VS and DS, Figure 4D). *Tt-FoxQ2 *expression is not found in the lateral extremities of the dorsal ectoderm. The expression of *Tt-FoxQ2 *in early trilobed larval stages is flanked by the expression of *Tt-Six 3/6 *and likely overlaps laterally in the dorsal ectoderm and within the deeper epithelial cells below. Later in larval development, *Tt-FoxQ2 *expression is restricted to a small subset of cells in the central portion of the anterior dorsal ectoderm (AE, Figure 4E) and one other small dorsal spot (DS, Figure 4E).

#### Tt-otp

Initially, *Tt*-*otp *is expressed in a large subset of cells throughout the animal cap ectoderm of the early radial gastrula (Figure 4F). This pattern is restricted to only a small subset of anterior dorsal cells near the midline of asymmetric middle gastrulae (Figure 4G) similar to the expression of *Tt-FoxQ2*. Bilateral late gastrulae express *Tt*-*otp *in a small number (10-15) of centrally located flask-shaped cells near the apical tuft region (FC, Figure 4H). At the early trilobed larval stage expression of *Tt*-*otp *is found in more numerous cells of the anterior dorsal ectoderm and a few cells on the ventral side of the mantle lobe (MC, Figure 4I). In later larval stages, *Tt*-*otp *is expressed in a subset of cells found in two bilaterally symmetric masses within the dorsal ectoderm and in a subset of cells on the ventral midline of the mantle lobe (DE and MC, respectively Figure 4J).

#### Tt-FoxG

*Tt*-*FoxG *is the only marker that is expressed in two distinct domains in the animal cap ectoderm of the early radial gastrula (AC, Figure 4K). Two additional, more lateral expression domains are added in asymmetric middle gastrulae (LC, Figure 4L), but expression of *Tt*-*FoxG *is not found along the embryo's anterior midline. Subsequently, the expression patterns of the two lateral-most domains (LC) expand in an equatorial ring around the middle of the apical lobe that correlates with the position of the developing transverse ciliated band and some rows of cells anterior to it (CB, Figure 4M). The two inner expression domains elongate (AC, Figure 4N) within the anterior ventral side of the apical lobe and connect to the equatorial ring of expression (CB, Figure 4N) on lateral sides of the remaining portion of the blastopore. *Tt-FoxG *is weakly expressed around the posterior edge of the small blastoporal opening of early trilobed larvae (Figure 4N). In late trilobed larvae, *Tt-FoxG *is generally expressed in a 'U'-shape domain that borders the anterior ventral ectoderm (VU, Figure 4O), and also within an anterior subset of the cells in the region of the transverse ciliated band (CB, Figure 4O).

#### Tt-Synaptotagmin 1

*Tt*-*synaptotagmin 1 *is expressed weakly in a small central area within the animal cap ectoderm of early radial stage gastrulae (Figure 4P). Asymmetric middle stage gastrulae express *Tt-synaptotagmin 1 *in a small central area of the dorsal ectoderm (CD, Figure 4Q). Bilateral late stage gastrulae maintain this expression domain and add two lateral expression domains that generally mark the ventral posterior border of the developing apical lobe of the larva (VP, Figure 4R). Expression of *Tt-synaptotagmin *1 is more pronounced at the early trilobed larval stage and is found in many of the cells of the developing anterior ectoderm (AE, Figure 4S), ectodermal cells positioned around the anterior tip of the foregut (EG, Figure 4S), and cells that border the ventral posterior edge of the apical lobe (VP, Figure 4S). These expression patterns are expanded in later larval stages as most (if not all) of the cells within the anterior dome region of the apical lobe express *Tt-synaptotagmin 1 *(AE, Figure 4T). Expression is maintained in ectodermal cells around the anterior portion of the foregut (EG, Figure 4T), and also within two equatorial ectodermal bands that generally mark the anterior and posterior edges of the transverse ciliated band (CA and CP, respectively Figure 4T).

## Discussion

### Axial partitioning of the larval neurogenic domains in *Terebratalia*

Despite the broad expression domains exhibited by genes such as *Tt-Six3/6, Tt-hbn*, and *Tt-NK2.1 *within the animal cap ectoderm at the early radial gastrula stage, subsequent morphological changes to the embryo during later stages of gastrulation clearly partition these expression domains into discrete dorsal and ventral regions. The remaining genes investigated here such as *Tt-fez, Tt-FoxQ2, Tt-otp*, and *Tt-FoxG *are expressed in different subsets of cells within the animal cap ectoderm of the radial gastrula stage and then shift either more dorsally or ventrally in a gene-specific manner. Based on these expression data and combined with the distribution of neuronal cells gathered from immunohistochemical preparations, there are at least two anterior neurogenic domains in the apical lobe of the larva (one dorsal and another ventral), as well as another neurogenic domain within the mid-ventral portion of the mantle lobe. Neuronal cells within the anterior dorsal domain include the broad sensory and supportive histaminergic epithelium of the apical organ [39], the ciliary photoreceptors [46], and the central neuropil. The anterior ventral neurogenic domain includes a wide cluster of basiepithelial histaminergic cells. Anti-histamine immunoreactivity is also found in the basiepithelial nerve rings that underlie the region of the transverse ciliated band [39]. *Tt-synaptotagmin 1 *expression is more pronounced in early and late trilobed larval stages consistent with this gene's role in late neuronal differentiation [49]. *Tt-synaptotagmin 1 *expression is mainly found in anterior neural tissues and the basiepithelial nerve rings of the transverse ciliated band.

**Figure 5:**
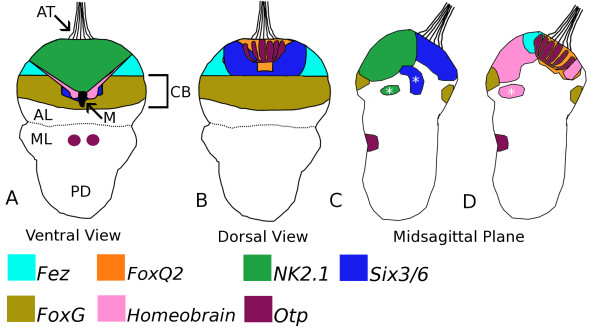
**Diagrams of ectodermal and endodermal (labeled with an asterisk) gene expression domains for *Terebratalia transversa *at the early trilobed larval stage**. All expression domains are based upon single probe *in situ *hybridizations with NBT/BCIP staining. The extent of domains and regions of overlap in expression among genes were inferred from the position of staining relative to morphological landmarks. The anterior ventral expression domains of *Tt-FoxG *are not depicted. Abbreviations: AL, apical lobe; AT, apical tuft; CB, region of the developing ciliated band; M, remaining portion of the blastopore; ML, mantle lobe; and PD, pedicle lobe.

The anterior ventral neurogenic domain is characterized by the broad expression of both *Tt-hbn *and *Tt-NK2.1*, both of which overlap with and are bordered laterally by *Tt-fez *in the anterior dorsal portion of the apical lobe. On the ventral surface of the apical lobe, both *Tt-hbn *and *Tt-NK2.1 *are bordered posteriorly by the expression of *Tt-FoxG *within the region of the transverse ciliated band (Figure 5A). The anterior dorsal neurogenic domain is largely demarcated by the expression of *Tt-Six3/6*, which overlaps laterally with *Tt-fez *expression (Figure 5B, C). The central portion of the dorsal neurogenic domain is delineated by *Tt-FoxQ2 *expression, where the cells that produce the apical ciliated tuft reside, but also includes the deeper epithelial cells within the apical organ. Also within this central zone are numerous flask-shaped surface epithelial cells that express *Tt-otp *(Figure 5D). Collectively the dorsal neurogenic domains encompass the cells that comprise the larval apical organ noted for numerous sensory neurons and deeper supportive epithelial cells, many of which express histamine [39]. Widespread distribution of histamine within the sensory cells (photoreceptors and statocysts) and peripheral nervous systems of a trematode flatworm, some mollusks, and arthropods is well documented [50-53], and collectively support a role for histamine as a modulator of muscular contractions and ciliary beat during locomotive behaviors. Altenburger *et al. *[38] found eight serotonergic sensory neurons in the larval apical organ of *T. transversa *that generally match the position and morphology of the central sensory neurons we labeled with the antibody against acetylated α-tubulin. The latter probe also recognizes approximately ten cells with specialized ciliary bundles within the ciliary tuft region at the late gastrula stage. These cells in *Terebratalia *are similar to the ampullary neurons described by Kempf and Page [54] from gastropod larvae, albeit the gastropods investigated consistently had only five ampullary neurons. Kempf and Page [54] also demonstrated that these five ampullary neurons were separate from the serotonergic sensory neurons within the larval apical organ. Since neurotransmitter expression has not been detected at the late gastrula stage in *Terebratalia*, it has not been possible to address this particular aspect of the apical organ's structure, but it should be noted that temporal separation of *otp *and neurotransmitter detection (serotonin) was also observed in similar larval cells of *Patella vulgata *[55]. By the trilobed larval stage *otp*-positive cells are present throughout much of the dorsal neurogenic domain and are likely beyond the limits of the *Tt-FoxQ2 *domain, overlapping with *Tt-fez *(Figure 5D).

Of the eight genes considered here, only *Tt-otp *is expressed in an anterior subset of cells within the mid-ventral neurogenic domain on the mantle lobe (Figure 5D). Although Stricker and Reed [48] identify this structure as a mid-ventral ciliated band, approximately thirty of the cells in this domain are histaminergic and are connected to the larval nervous system [39]. The mid-ventral neurogenic domain also exhibits the greatest degree of centralization, and coupled with the expression of *Tt-otp *supports the interpretation that some neural cell types in the mid-ventral (mantle) neurogenic domain and the ventral nerve cord of annelids are conserved [56]. However, since the anterior ventral neurogenic domain in the apical lobe of *Terebratalia *larvae is not centralized, then aspects of both anterior-posterior patterning and centralization of neurogenic domains may be different between *Platynereis *and *Terebratalia*. Collectively, however, these gene expression and immunohistochemical domains in *T. transversa *larvae are still reminiscent of anterior and ventral neurogenic domains in spiralian larval forms (apical organ, cerebral ganglion, and ganglionated ventral nerve cord), albeit complicated by the basiepithelial nature of the larval nervous system in *T. transversa*. To evaluate the putative homology of these neurogenic domains we review the known expression patterns for genes we have isolated in *T. transversa *in several larval forms among protostome and deuterostome animals in the following sections.

### Comparisons among embryos and larval types

**Figure 6:**
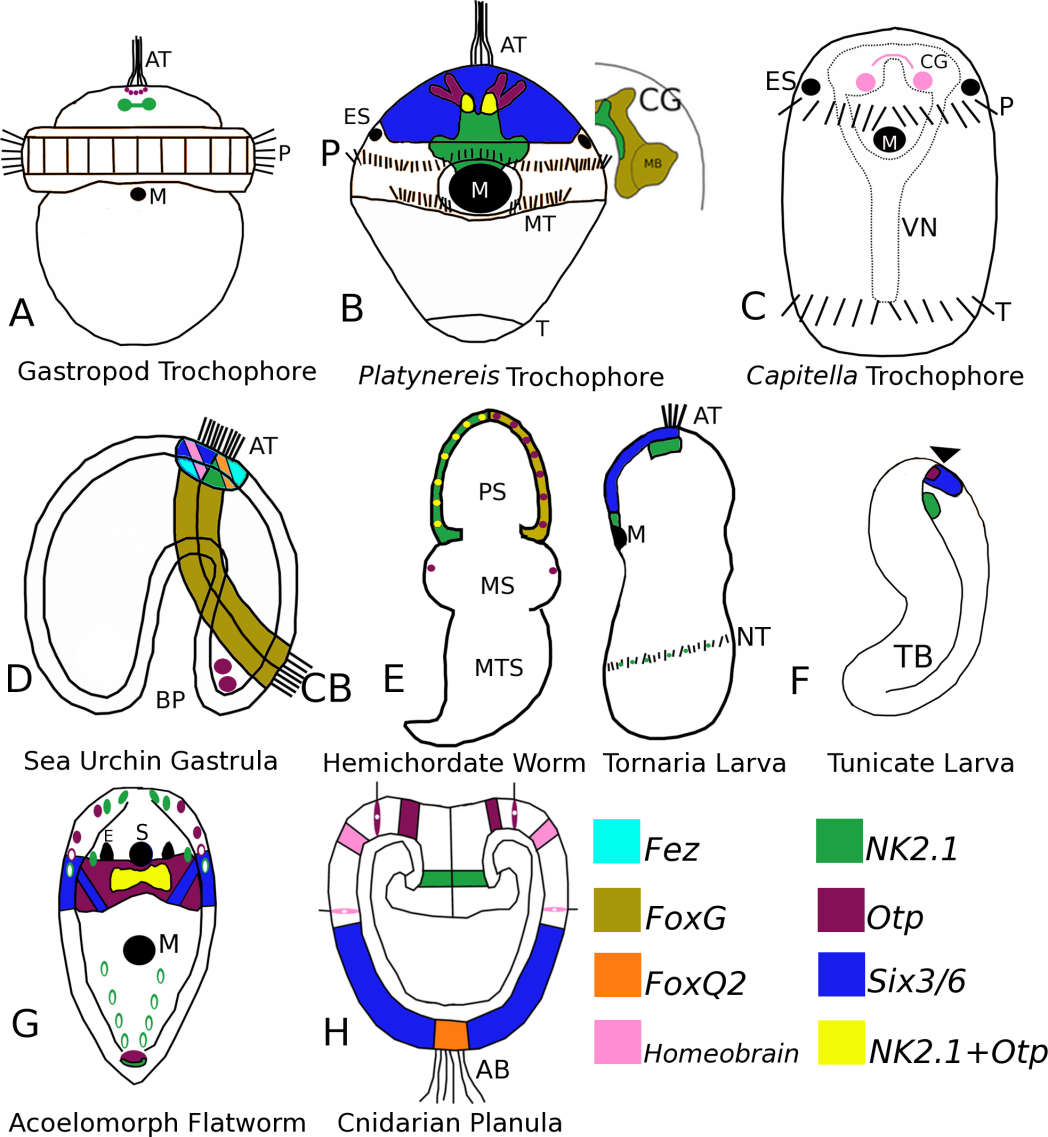
**Composite expression domains for orthologous genes involved in the patterning of neural and other ectodermal tissues from developmental stages, larval types, or adult forms of representative invertebrate animals**. Figure panels based on [5,14-16,41-45,55,56,60-63,66,68,70,71,85-87], see text for details. Abbreviations: AB, aboral tuft of cilia; AL, apical lobe; AT, apical tuft; BP, blastopore; CB, ciliated band; CG, cerebral ganglia; E, adult eyespot; ES, larval eyespot; M, mouth; MB, mushroom body; ML, mantle lobe; MS, mesosome; MT, metatroch; MTS, metasome; NT, neotroch; P, prototroch; PL, pedicle lobe; S, statocyst; T, telotroch; TB, tailbud; VN, ventral nerve cord.

Species-specific tissue types, differences in developmental stages, and derived anatomical features of particular larval types complicate comparisons of gene expression patterns among disparate animals. In terms of the developing nervous system, discerning discrete larval versus juvenile expression domains can be difficult in more gradually developing animals such as annelids and mollusks, as opposed to echinoderms in which dramatic distinctions between larval and adult structures can be made [57-59]. However, there are particular anatomical features of embryological stages and larvae among spiralians and deuterostomes that can be used to minimize the amount of developmental variation in comparative datasets. We therefore focused on the expression of these genes during the late gastrula-early larval transition stage when aspects of the developing larval nervous system and, in particular, anterior neurogenic domains associated with the larval apical organ are present. To these ends, we review the expression of the genes we have isolated for *Terebratalia *to orthologous genes in similar developmental stages of various bilaterian animals in Figure 6.

### Spiralians

Expression data for these genes from molluscan trochophores are not well characterized, except for *otp *and *NK2.1*. In the trochophore larva of the limpet, *Patella vulgata, Pv-otp *is expressed in a 'U'-shaped field of ectodermal cells that surrounds the apical tuft region (Figure 6A). These cells correspond to the position of serotonergic flask-shaped neurons within the apical organ, but dual labeling was not possible since *Pv-otp *was expressed before the neurotransmitter type of these cells was detectable [55]. The trochophore larva of the abalone, *Haliotus rufescens*, expresses *Hr-NK2.1 *in the developing cerebral ganglion and not in the larval apical organ [41]. Anterior neurogenic domains are better characterized in the trochophore larva of the polychaete annelid, *Platynereis dumerilii*. In this species, *Pd-Six3/6 *also demarcates a broad anterior neurogenic tissue domain [44], but *Pd-NK2.1 *is limited to a more centralized anterior region (Figure 6B) mainly within the developing cerebral ganglia, and partially overlaps with *Pd-otp *expression in extraocular vasotocinergic photoreceptors [42]. It is not clear whether or not the more anterior cells that express *Pd-otp*, but not *Pd-NK2.1*, are positioned within the larval apical organ of *Platynereis*. The developing cerebral ganglia in *Platynereis dumerilii *larvae generally express *Pd-FoxG *(*BF-1*) in proximity to the expression of *Pd-NK2.1 *and especially within the structures that Tomer *et al. *[60] called mushroom bodies (MB, Figure 6B). *Homeobrain-like *expression is known for another polychaete annelid, *Capitella teleta*, where *Ct-hbn *is expressed in a subset of cells in the developing cerebral ganglia (Figure 6C, see [61]). Collectively, these data support the hypothesis that the spiralian larval apical organ is a *Six3/6*-dependent neurogenic domain that contains *otp*-positive neurons. Both of these features are also present in *T. transversa *larvae, supporting the idea that both brachiopods and spiralians share an ancestor with a larva that contained such an organ. The spiralian cerebral ganglion generally expresses *FoxG *and *NK2.1*, and, at the very least, some cells that also express *homeobrain*. Although both *Tt-hbn *and *Tt-NK2.1 *are broadly expressed in the anterior neurogenic domains of the early gastrulation stages, their final expression state within *Terebratalia *larvae suggests that only the anterior ventral neurogenic domain is homologous to the spiralian cerebral ganglion. However, since *Tt-FoxG *is expressed in cells that contribute to the deeper epithelial cells within the apical organ, it is possible that both the deeper apical organ cells (nonsensory types) and the histaminergic cells within the ventral cluster in the apical lobe of the larva are homologous to the cerebral ganglion of spiralians.

### Deuterostomes

The developing apical plate in the late gastrulae of sea urchins (Figure 6D) is a *Six3/6 *dependent neurogenic domain [62] that also broadly expresses *NK2.1, homeobrain*, and *FoxQ2 *[45,63,64]. Although *otp *is a marker for specific neuronal cell types in deuterostomes, trochozoans, and ecdysozoans [65], its role in the gastrulation of echinoderms is mainly linked to the differentiation of skeletalogenic mesenchyme or oral ectodermal cell fates [66,67]. *FoxG *is expressed throughout the oral ectoderm in the blastula, but is restricted to the developing ciliated bands during gastrulation [68]. Recent work in the urchin, *Hemicentrotus pulcherrimus*, suggests that *Hp-fez *is positively regulated by *Hp-FoxQ2*, and that the fez protein serves to maintain the size of the neurogenic animal ectoderm by modulating the inhibitory effects of *BMP2/4 *[69]. Our results for *Tt-fez *are consistent with these observations as *Tt-fez *expression generally borders the *Tt-FoxQ2 *domain. *Six3*/*6 *also demarcates the anterior ectoderm in the development of the direct-developing hemichordate worm, *Saccoglossus kowalevskii *([43]; Figure 6E). Expression patterns of the remaining genes are better known from the developing juvenile where both *Sk-NK2.1 *and *Sk-FoxG *are expressed in ectoderm of the prososome region. *Sk-otp *is also co-expressed in this region, but only in a punctate pattern of select ectodermal cell types [43]. Expression of *NK2.1 *in a hemichordate that exhibits a primary larval developmental pattern (*Ptychodera flava*) is found in the developing apical organ, the anterior lip of the mouth, and within select neuronal cells of the neotroch ([70]; Figure 6E). Expression of *otp *and *FoxG *within the larval tissues of *P. flava *has not been published. In the tail bud stage of the tunicate, *Ciona intestinalis, Ci-Six3*/*6 *is expressed anteriorly near the stomodeum and within the sensory vesicle ([71]; Figure 6F). *Ci-otp *is also expressed by a select group of cells within the *Ci-Six3*/*6 *domain, but these cells do not co-express *Ci-NK2.1. Ci-NK2.1 *is expressed in two bilaterally symmetrical patches of cells on the anterior-ventral side of the developing neural tube [71]. Overall, the expression patterns for these genes in deuterostomes are more diverse than those reported among the spiralian animals available; however, the anterior larval neural ectoderm is a *Six3/6*-dependent domain suggesting that this feature is plesiomorphic for all bilaterians [44], and that it may include a central group of *otp*-positive neurons.

### Acoelomorphs

Insight into the complexity and degree of centralization of the adult nervous system of the ancestor of protostomes and deuterostomes may be gained from studying the development of acoelomorph 'flatworms' [5], depending on their true phylogenetic position [8,25]. The majority of evidence supports the idea that the gene networks that control the patterning of anterior ectoderm in bilaterian larvae were co-opted from a direct-developing ancestor [5,72] and ancient gene networks controlling oral ectodermal cell fates [15]. It therefore remains plausible that comparing the expression of the genes we include here for *Terebratalia *to orthologous genes in direct-developing acoelomorph flatworms may yield insights into their plesiomorphic role in bilaterians. In the developing juvenile of the acoel, *Convolutriloba longifissura, Cl-Six3/6, Cl-otp*, and *Cl-NK2.1 *are all expressed in the nervous system, particularly in the compact anterior 'brain' [5,6]. Within this neurogenic domain, *Cl-Six3/6 *is expressed generally within the anterior neural ectoderm, and the expression domains of *Cl-otp *and *Cl-NK2.1 *are found more centrally within it ([5]; see Figure 6G). Both *Cl-NK2.1 *and *Cl-otp *are also expressed in putative sensory cells anterior to the brain, as well as in sensory cells on the ventral side of the body ([5]; unfilled circles see Figure 6G). Based on these expression patterns in acoels, combined with what is known for both spiralians and deuterostomes, it is likely that the ancestor of protostomes and deuterostomes had a single anterior neurogenic domain that expressed (at least) *Six3/6, otp*, and *NK2.1*, but *homeobrain *and *fez *domains are also likely. Subsets of these neurogenic fields (such as *Six3/6 *+ *otp *and *NK2*.1 + *homeobrain*) are progressively separated into dorsal and ventral domains during the gastrulation of true spiralians and other related taxa with trochozoan larval features such as *T. transversa*. Since *Cl-otp *is also expressed in a subset of ventral neural cells similar to *Tt-otp*, and these expression patterns are spatially similar to what is observed in the mesosome of the hemichordate worm, *Saccoglossus kowalevskii *[43], a more posterior ventral neurogenic domain may also be plesiomorphic for bilaterians. This hypothesis is also supported by the more ventral position of *NK2.1 *expression in the neural tube of the tunicate, *C. intestinalis *[71] as well as the expression of other *NK*-class genes in the ventral nerve cord of spiralians [2]. Since the origin of these gene families predates the Bilateria [73,74], a discussion of their role in cnidarian and ctenophore development is useful as it relates to the origin of bilaterian larval apical organs.

### Origin and diversification of larval apical organs

The cellular morphology of the bilaterian anterior larval nervous systems may include an apical organ consisting of a single group or bilateral masses of numerous (thirty of more) bipolar serotonergic neurons, some of which are sensory, as found in the larvae of particular classes of echinoderms and hemichordates [75-77]. Spiralian larval apical organs usually consist of four to eight central serotonergic sensory neurons surrounded by nonsensory peripheral neurons [78]. Clusters of neuronal cells that all express the same neurotransmitter type in ambulacralian deuterostomes contributed to the description of these structures as larval 'ganglia' [18]. However the plesiomorphic state of ambulacralian apical organs may have only been a simple bilaterally symmetric nerve plexus formed by two groups of neuronal cell bodies that originate from opposing ciliary band regions [76]. The larval apical organs of entoprocts, annelids, and mollusks are usually limited to four to eight central serotonergic flask-shaped cells and some peripheral serotonergic neurons that also express FMRFamide (Phe-Met-Arg-Phe-NH_2_), none of which originate from the larval ciliary bands [78-81]. Although the individual neurotransmitter cell types among ambulacralian deuterostomes, annelids, and mollusks appear similar in morphology, establishing definitive homologous cell types remains problematic, and these cells are deployed in what could be independently derived apical neural structures.

Our results of the expression of *Tt-otp *within a subset of cells of the developing apical organ of late gastrula stage *Terebratalia *show striking similarities to the morphology of the central (serotonergic) flask cells of larval entoprocts, annelids, and some mollusks [78-81], supporting the hypothesis that these animals share an ancestry that included a larval form that contained such an organ. This hypothesis is also supported by the presence of ampullary-like neurons within the apical tuft region similar to those described for gastropod mollusks [54]. However, spiralian larval morphology and apical organs generally reflect the anatomy of the late gastrula stage [59], as indicated by the early developmental expression of particular neurotransmitter types and the relatively fewer number of neuronal cells within their apical organs [78]. Larval development in *Terebratalia *coincides with late differentiation of neuronal and other ciliated cell types (*otp*-positive cells, ampullary neurons, or other). This interpretation is supported by numerous flask-shaped cells that express *Tt-otp *in the apical organ of the trilobed larval stage and also correlates with the widening of the *Tt-FoxQ2 *domain at this stage. Expression of *Tt-Six3/*6, *Tt-fez, Tt-FoxQ2*, and *Tt-otp *in the dorsal neural ectoderm that gives rise to the apical organ continues into the late larval stage. These expression patterns are also reflected in the numerous sensory neurons and other supportive cells labeled by anti-acetylated α-tubulin and anti-histamine only at the late larval stage.

Some aspects of ciliary tuft development predate the Bilateria, as orthologs of *FoxQ2, COE*, and *FGFa1 *are expressed within the region of the aboral ciliary tuft in planula larvae of the hydrozoan, *Clytia hemisphaerica*, and the anthozoan, *Nematostella vectensis *[15,82,83]. Based on the spatial expression of numerous genes involved in the axial patterning of bilaterian animals within *Nematostella *planulae, the oral-aboral axis of cnidarians is believed to be homologous to the anterior-posterior axis in bilaterians, and the bilaterian dorsal-ventral axis is homologous to the cnidarian directive axis [84]. The oral pole of cnidarians contains *NK2.1, homeobrain, noggin1*, and *otp *expression domains and several other bilaterian anterior-related genes [16,85] except *Six 3/6 *that is present in the aboral region of the larva ([86,87]; see Figure 6H). One possible reason behind the aboral position of *Six3/6 *expression in planulae may be because the oral pole in cnidarians is the site of gastrulation where canonical *WNT *signaling specifies endodermal tissues and has inhibitory effects on the specification of neural ectoderm [88]. Although not characterized in cnidarian planulae, *FoxG *expression is known for the direct-developing ctenophore, *Mnemiopsis leidyi *(*cteno-BF1*, [89]) where it is expressed in the developing tentacle buds that flank the aboral organ. Taken together, there are at least two different ectodermal domains in planulae, the aboral region that includes the ancient ciliary tuft characterized by *Six3/6, FoxQ2*, and possibly *FoxG *expression domains and an oral region with particular neuronal cell types characterized by (at least) *homeobrain, otp*, and *NK2.1 *expression. How these separate expression domains became coupled at the animal pole of bilaterian embryos, the adult head, and the bilaterian larval apical organ remains unclear (but see [10] for a plausible hypothesis).

## Conclusions

Although wide scale homology may be present in select, centralized, apical neuronal cell types among evolutionarily distant larval types [39], species-specific deployment of these cell types within the anterior regions of diverse larval forms may result in independently derived apical organs (such as in nemerteans, see [90]), evolutionarily old cell types may be used in new ways [46], or specific developmental patterns may result in the partial or complete absence of these cellular features. As *T. transversa *larvae are of a primary larval type with a cataclysmic metamorphosis it will be interesting to compare these gene expression patterns in the ventral ganglion of the adult form [91], within similar structures in direct-developing species such as *Glottidia *[39], as well as within larval phoronids that exhibit intriguing patterns in the development of larval and juvenile traits [35]. Overall, our data support the conclusion that modifications to the combinatorial expression patterns of the genes we include here account for a significant amount of the cytological variation in bilaterian larval apical organs. Based on corresponding expression domains gathered from various larval and adult bilaterians it is likely that the plesiomorphic state of the anterior bilaterian nervous system included a single compact mass of neurons, subsets of which expressed *Six3/*6, *otp, NK2.1, hbn, fez*, and *FoxQ2*. Testing this hypothesis further requires similar expression data from broader taxonomic groups. Future work should also focus on genes involved in either restricting or expanding the anterior neurogenic domains such as *BMP2/4 *and *delta-Notch *signaling [69,92] to discern their role in the diversification of larval and adult nervous systems.

## Methods

### Adult collection and larval cultures

Adults of *Terebratalia transversa *(Sowerby, 1846) were dredged from the waters adjacent to San Juan Island, Washington, USA during the early spring of 2005 and the winter of 2008. Adults were maintained in a free-flowing seawater table (8 to 10°C) at Friday Harbor Laboratories until needed. Gametes were gathered and larval cultures were created using the methods of Reed [93]. Briefly, eggs were stripped from the gonads of ripe females and sieved through a 250-μm Nitex mesh into 200 ml 0.45 μm-filtered seawater. Eggs harvested this way still have an intact germinal vesicle that will break down and a layer of follicle cells that will be shed if left in seawater for three to four hours, after which the eggs can be fertilized. The testes of ripe males were harvested similarly except that 2 to 3 ml of concentrated sperm were diluted into a 50 ml beaker containing 0.45 μm-filtered seawater and then this solution was brought to a final pH of 9.8 using 1 N NaOH. This process activated the sperm and approximately 5 ml of this solution was used to fertilize the eggs. Fertilized cultures were then washed with filtered seawater after one hour. Embryological and larval cultures were maintained at ambient seawater temperatures in a seawater table in glass bowls and the seawater was changed daily.

### Gene isolation

*Tt-fez, Tt-FoxG, FoxQ2, Tt-homeobrain, Tt-NK2.1 *and *Tt-otp*, were identified from Expressed Sequence Tags (ESTs) clones sequenced for a previous phylogenomic analysis [94] publicly available on dbEST NCBI (National Center for Biotechnology Information; http://www.ncbi.nlm.nih.gov/projects/dbEST/). Fragments of *Tt-synaptotagmin 1 *and *Tt-Six3/6 *were amplified by degenerate PCR using cDNA generated from mRNA isolated from mixed embryological stages. Nested primer sets for *Tt-Six3/6 *were *Six3/6 *F1 FLSWSLP 5' TTYYTNTGGWSNYTNCC 3', *Six3/6 *R1 QRDRAA 5'GCNGCNCKRTCNCKYTG 3', *Six3/6 *R2 NWFKNRRQ 5'TGNCKNCKRTTYTTRAACCARTT 3', *Six3/6 *F2 GPVDKYRV 5'GGNCCNGTNGAYAARTAYMGNGT 3'. Nested primer sets for *Tt-synaptotagmin *were Syn F1: 5' TYAAYCCNGTNTTYAAYGA 3', Syn F2: 5' TAYGAYTTYGAYMG/ideoxyI/TT 3', Syn R1: 5' TCRTTRTARTANGGRTT 3', Syn R2: 5' SWRAARCADATRTC/ideoxyI/CC 3'. Full-length cDNAs were obtained by rapid amplification of cDNA ends using the SMART RACE kit (Clontech Laboratories, Inc., Mountain View, CA, USA) using sequence specific primers.

### Phylogenetic analyses

The deduced amino acid sequences for *Terebratalia forebrain embryonic zinc-finger, Forkhead G, orthopedia, otx, NK2.1, homeobrain, synaptotagmin 1*, and *Six3/6 *along with those for representative related proteins from other taxa, retrieved from NCBI (html://http://ncbi.nlm.nih.gov/; accession numbers listed below) and Joint Genome Institute (http://genome.jgi-psf.org/Capca1/Capca1.home.html for *Capitella teleta*; http://genome.jgi-psf.org/Lotgi1/Lotgi1.home.html for *Lottia gigantea*; http://genome.jgi-psf.org/Nemve1/Nemve1.home.html for *Nematostella vectensis*; http://genome.jgi-psf.org/Triad1/Triad1.home.html for *Trichoplax adhaerens*; protein ID numbers listed below), were aligned with MUSCLE [95] or MacVector. The resulting alignments of conserved domains were corrected by eye. For each dataset, Bayesian phylogenetic analysis was performed using a parallelized version of MrBayes [96,97], with four independent runs and a mixed model of protein evolution. Each analysis was run until the average standard deviation of split frequencies between runs was less than 0.01 (10,000,000 generations for fez, NK-class, Paired-class, Six-class and Synaptotagmin datasets; 20,000,000 generations for the Fox-class dataset). A consensus tree and posterior probabilities for each node were calculated from the final 2,000,000 generations of each run. For all accession numbers and protein sequences used in phylogenetic analyses see additional files.

### Whole-mount *in situ *hybridization

*In situ *hybridizations were conducted using an established protocol [98]. Probes were synthesized with dUTP-digoxigenin (Roche Applied Science, Indianapolis, IN, USA) and hybridized at a concentration of 1 ng/μl at 63°C for 48 hours. The hybridization buffer contained 50% formamide, 5× sodium citrate buffer, 50 μg/ml heparin, 0.1% Tween-20, 1% SDS, and 100 μg/ml denatured salmon sperm DNA. Probes were detected with anti-digoxigenin-AP antibody at 1:5000 in blocking buffer (Roche Applied Science, Indianapolis, IN, USA), and subsequently visualized with 330 ng/ml nitroblue tetrazolium chloride (NBT) and 165 ng/ml 5-bromo-4-chloro-3-indolyl phosphate (BCIP) (Roche Applied Science, Indianapolis, IN, USA).

### Immunohistochemistry

Fixation and immunohistochemical procedures of embryos and larvae followed Santagata [39]. Briefly, specimens were fixed either for 20 minutes or overnight at 4°C in a 4% paraformaldehyde solution in 0.1 M Sørenson's phosphate buffer (pH *= *7.4) or filtered seawater. Larvae were removed from this solution and larval tissues were permeablized with 0.1 M Sørenson's phosphate buffer and 0.1% to 0.3% Triton-X detergent (PTA solution) for 24 hours at 4°C before proceeding with the staining protocol. All further steps were carried out on a rotary shaker table. Nonspecific sites were blocked with 4% normal goat serum (S-1000, Vector Laboratories, Burlingame, CA, USA) in PTA solution (GS-PTA) for 24 hours at 4°C. The primary antibody (anti-acetylated α-tubulin, T-6793, Sigma-Aldrich, St. Louis, MO, USA) or anti-histamine (Immunostar, Hudson, WI, USA) was diluted 1:500 with 4% GS-PTA and incubated with the larvae for 48 hours at 4°C. Specimens were washed (three times) in PTA for a total of 24 hours. Larvae were exposed to the secondary antibody, goat anti-mouse or rabbit AlexaFluor 488 or 568 immunoglobin (A21121, A21124, A11008, or A11011, Invitrogen-Molecular Probes, Eugene, OR, USA), at a dilution of 1:50 or 1:100 for 24 hours. Deleting the primary antibody from the protocol produced negative controls. Some specimens were then stained for either fibrous actin or nucleic acids. Nucleic acids were stained with a 1:1,000 dilution of sytox green (S7020, Invitrogen-Molecular Probes) for 10 minutes or a 1:500 dilution of propidium iodide (P-1304, Invitrogen-Molecular Probes) for 10 to 15 minutes. Specimens were adhered to clean glass slides coated with a poly-L-lysine solution (1:10 dilution, 25988-63-0, Sigma-Aldrich) and put through an alcohol dehydration series using 2-propanol within four minutes. Finally, specimens were cleared in a solution of benzyl benzoate and benzyl alcohol (2:1) for two minutes and mounted in the same solution. Slides were kept in the dark at 4°C until viewed with a BioRad Radiance 2100 laser confocal system and a Nikon E800 microscope (Friday Harbor Laboratories, Friday Harbor, WA, USA) or a Zeiss 710 Confocal Laser Scanning Microscope (Cold Spring Harbor Laboratory, NY, USA). Confocal z-series were gathered as 1 μm sections. Z-projections and volume renderings of embryos and larvae were created using OsiriX (Pixmeo, Switzerland) or FIJI.

### Accession numbers for sequences included in phylogenetic analyses

#### Fez

*Branchiostoma *Fez (ADK13096.1); *Caenorhabditis *Y38H8A.5 (NP_502594.2); *Capitella *fez (18104); *Capitella *Gfi (45287); *Drosophila *earmuff (NP_608631.1); *Daphnia *fez (EFX89329.1); *Homo *fez1 (NP_001019784.2); *Homo *fez2 (NP_060478.3); *Homo *Gfi-1b (NP_004179.3); *Homo *ZF430 (AAP30885.1); *Homo *ZF85 (NP_003420.20; *Lottia *fez (68213); *Lottia *Gfi1 (129344); *Lottia *Gfi2 (83709); *Mus *fez1 (NP_082738.1); *Mus *fez2 (NP_536681.2); *Nematostella *12000017 (228271); *Nematostella *265000001 (230810); *Nematostella *30000108 (201757); *Nematostella *e_gw.3.372.1 (80425); *Nematostella *Gfi (182742); *Saccoglossus *fez (NP_001158457.1); *Schmidtea *fez (XP_002575460.1); *Schmidtea *Gfi (XP_002580588.1); *Trichoplax *fez (7089); *Trichoplax *Gfi (63664); *Terebratalia *fez (JQ88195)

#### Fox-class

*Clytia *FoxQa (ABG21224.1); *Clytia *FoxQb (ABG21225.1); *Capitella *FoxA (169665); *Capitella *FoxAB (131123); *Capitella *FoxB (225366); *Capitella *FoxC (199610); *Capitella *FoxD (126386); *Capitella *FoxF (50240); *Capitella *FoxG (139421); *Capitella *FoxGa (182306); *Capitella *FoxI (154409); *Capitella *FoxJ1 (222987); *Capitella *FoxJ2/3 (137131); *Capitella *FoxK (23732); *Capitella *FoxL1 (49410); *Capitella *FoxL2 (88179); *Capitella *FoxM (115253); *Capitella *FoxN1/4 (129521); *Capitella *FoxN2/3 (102038); *Capitella *FoxO (91312); *Capitella *FoxP (173180); *Capitella *FoxQ1 (175391); *Capitella *FoxQ2a (111555); *Capitella *FoxQ2b (148596); *Nematostella *(110212); *Nematostella *(118122); *Nematostella *(120142); *Nematostella *(121754); *Nematostella *(123903); *Nematostella *(125256); *Nematostella *(132285); *Nematostella *(138488); *Nematostella *(150900); *Nematostella *(161006); *Nematostella *(165261); *Nematostella *(165603); *Nematostella *(18324); *Nematostella *(187332); *Nematostella *(192525); *Nematostella *(19405); *Nematostella *(200222); *Nematostella *(200356); *Nematostella *(201028); *Nematostella *(213966); *Nematostella *(218419); *Nematostella *(228732); *Nematostella *(38679); *Nematostella *(39596); *Nematostella *(39632); *Nematostella *(5001); *Nematostella *(58039); *Nematostella *(59063); *Nematostella *(65438); *Nematostella *(67043); *Nematostella *(67209); *Nematostella *(88569); *Nematostella *(93177); *Nematostella *(96685); *Lottia *FoxA (183845); *Lottia *FoxAB (99760); *Lottia *FoxB (186344); *Lottia *FoxC (117369); *Lottia *FoxD (137594); *Lottia *FoxF (117350); *Lottia *FoxG (59807); *Lottia *FoxH (134143); *Lottia *FoxJ1 (69660); *Lottia *FoxJ1 (59864); *Lottia *FoxJ2/3 (98413); *Lottia *FoxK (183124); *Lottia *FoxL1 (178394); *Lottia *FoxL2 (89841); *Lottia *FoxN2/3 (138633); *Lottia *FoxP (54435); *Lottia *FoxQ2 (79770); *Mus *FoxA1 (NP_032285.2); *Mus *FoxA2 (NP_034576.2); *Mus *FoxB1 (NP_071773.2); *Mus *FoxB2 (NP_032049.1); *Mus *FoxC1 (NP_032618.2); *Mus *FoxC2 (NP_038547.2); *Mus *FoxD2 (NP_032619.1); *Mus *FoxD3 (NP_034555.3); *Mus *FoxD4 (NP_032048.1); *Mus *FoxE1 (NP_899121.1); *Mus *FoxE3 (NP_056573.1); *Mus *FoxF1 (NP_034556.1); *Mus *FoxF2 (NP_034355.2); *Mus *FoxG1 (NP_032267.1); *Mus *FoxH1 (NP_032015.1); *Mus *FoxI1 (NP_076396.3); *Mus *FoxI2 (NP_899016.1); *Mus *FoxJ1 (NP_032266.3); *Mus *FoxJ2 (NP_068699.1); *Mus *FoxJ3 (NP_766287.1); *Mus *FoxK1 (NP_951031.2); *Mus *FoxK2 (NP_001074401.2); *Mus *FoxM1 (NP_032047.4); *Mus *FoxN1 (NP_032264.1); *Mus *FoxN2 (NP_851305.2); *Mus *FoxN4 (NP_683737.2); *Mus *FoxO1 (NP_062713.2); *Mus *FoxO3 (NP_062714.1); *Mus *FoxO6 (NP_918949.1); *Mus *FoxP1 (NP_444432.1); *Mus *FoxP2 (NP_997600.1); *Mus *FoxP3 (NP_473380.1); *Mus *FoxP4 (NP_083043.2); *Mus *FoxQ1 (NP_032265.3); *Mus *FoxS1 (NP_034356.1); *Platynereis *FoxG (ADG26725.1); *Ptychodera *FoxQ2 (ADZ61650.1); *Rattus *FoxR1 (XP_243815.4); *Rattus *FoxR2 (XP_228808.3); *Strongylocentrotus *FoxI (ABB89485.1); *Strongylocentrotus *Fox_L1 (ABB89488.1); *Strongylocentrotus *FoxA (ABE68834.1); *Strongylocentrotus *FoxAB-like (ABB89474.1); *Strongylocentrotus *FoxB (NP_999797.1); *Strongylocentrotus *FoxC (ABB89478.1); *Strongylocentrotus *FoxD (ABB89476.1); *Strongylocentrotus *FoxF (ABB89479.1); *Strongylocentrotus *FoxG (ABB89477.1); *Strongylocentrotus *FoxJ1 (ABB89480.1); *Strongylocentrotus *FoxK (ABB89486.1); *Strongylocentrotus *FoxL2 (ABB89483.1); *Strongylocentrotus *FoxM (ABB89490.1); *Strongylocentrotus *FoxN1/4 (ABB89491.1); *Strongylocentrotus *FoxN2/3 (ABB89482.1); *Strongylocentrotus *FoxO (ABB89484.1); *Strongylocentrotus *FoxP (ABB89487.1); *Strongylocentrotus *FoxQ1 (ABB89489.1); *Strongylocentrotus *FoxQ2 (ABB89473.1); *Terebratalia *FoxG (JQ88193); *Terebratalia *FoxQ2 (JQ88200)

#### NK-class

*Capitella *Lbx (ACI26672.1); *Capitella *NK-like-1a (ACH70609.1); *Capitella *NK-like-1b (ACI26669.1); *Capitella *NK-like-2.1a (ACH89430.1); *Capitella *NK-like-2.1b (ACH89431.1); *Capitella *NK-like-2.2a (ACH89432.1); *Capitella *NK-like-2.2b (ACH89433.1); *Capitella *NK-like-3 (ACI26670.1); *Capitella *NK-like-4a (ACH89434.1); *Capitella *NK-like-4b (ACH89435.1); *Capitella *NK-like-5 (ACH89437.1); *Capitella *NK-like-5b (ACH88440.1); *Capitella *NK-like-6 (ACI26668.1); *Capitella *NK-like-7_ACI26671.1); *Capitella *Tlx (ACH89436.1); *Drosophila *bap (NP_732637.1); *Drosophila *C15 (NP_476873.2); *Drosophila *dll (NP_726486.1); *Drosophila *drop (NP_477324.1); *Drosophila *H6 (NP_732244.3); *Drosophila *lbe (NP_524435.2); *Drosophila *lbl (NP_524434.2); *Drosophila *scro (NP_001015473.1); *Drosophila *slou (NP_476657.1); *Drosophila *tinman (NP_524433.1); *Drosophila *vnd (NP_001036253.1); *Mus *Dlx1 (NP_034183.1); *Mus *Dlx2 (NP_034184.1); *Mus *Dlx3 (NP_034185.1); *Mus *Dlx4 (NP_031893.3); *Mus *Dlx5 (NP_034186.2); *Mus *Dlx6 (NP_034187.1); *Mus *HMX1 (NP_034575.1); *Mus *HMX2 (NP_666110.1); *Mus *HMX3 (NP_032283.3); *Mus *Lbx1 (NP_034821.2); *Mus *Lbx2 (NP_034822.1); *Mus *Msx1 (NP_034965.2); *Mus *Msx2 (NP_038629.2); *Mus *Msx3 (NP_034966.1); *Mus *Nk2.5 (NP_032726.1); *Mus *Nk3.2 (NP_031550.2); *Mus *Nkx-3.1 (NP_035051.1); *Mus *Nkx-6.1 (NP_659204.1); *Mus *Nkx-6.2 (NP_899071.2); *Mus *Nkx-6.3 (NP_083278.1); *Mus *Nkx1.1 (XP_001473685.1); *Mus *Nkx1.2 (NP_033149.1); *Mus *Nkx2.1 (NP_033411.3); *Mus *Nkx2.2 (NP_035049.1); *Mus *Nkx2.3 (NP_032725.1); *Mus *Nkx2.4 (NP_075993.1); *Mus *Nkx2.6 (NP_035050.2); *Mus *Nkx2.8 (NP_032727.2); *Mus *Tlx1 (NP_068701.1); *Mus *Tlx2 (NP_033418.1); *Mus *Tlx3 (NP_064300.2); *Platynereis *Dlx (CAJ38799.1); *Platynereis *Lbx (ABQ10642.1); *Platynereis *Msx (CAJ38810.1); *Platynereis *Nk1 (CAJ38797.1); *Platynereis *NK2.1 (CAJ38809.1); *Platynereis *NK2.2 (ABO93209.1); *Platynereis *NK3 (ABQ10641.1); *Platynereis *NK4 (ABQ10640.1); *Platynereis *NK5 (ABQ10644.1); *Platynereis *Tlx (ABQ10643.1); *Terebratalia *NK2.1 (JQ88197)

#### Paired-class

*Crepidula *Pitx (ADI48168); *Ciona *Prop (XP (002119699); *Ciona *otp (NP (001072023); *Ciona *otx (NP (001027662); *Capitella *EBX (ABC58683); *Capitella *Gsx (AAZ23124); *Capitella *Pax3-7 (ABC68267); *Capitella *Xlox (AAZ95509); *Capitella *cdx (AAZ95508); *Drosophila *Pitx (NP (733410); *Drosophila *Vsx1 (NP (572232); *Drosophila *Vsx2 (NP (001033832); *Drosophila *aristaless (NP (722629); *Drosophila *dll (NP (523857); *Drosophila *gsc (NP (476949); *Drosophila *homeobrain (NP (788420); *Drosophila *otd (P22810); *Drosophila *otp (P56672); *Drosophila *repo (NP (477026); *Drosophila *rx (NP (726006); *Hydroides *otx (ABK76302); *Platynereis *Arx (ADG26723); *Platynereis *Cdx (ACH87546); *Platynereis *Gsx (ACH87540); *Platynereis *Pax6 (CAJ40659); *Platynereis *Xlox (ACH87551); *Platynereis *dlx (CAJ38799); *Platynereis *dlx (CAJ387991); *Platynereis *gsc (CAC19336); *Platynereis *otp (ABR68849); *Platynereis *otx (CAC19028); *Platynereis *rx (AAU20320); *Patella *gsc (CAD45551); *Patella *otp (AAM33145); *Patella *otx (AAM33144); *Saccoglossus *Prop (NP (001161635); *Saccoglossus *hbn (XP (002731203); *Saccoglossus *otd (NP (001158360); *Saccoglossus *otp (NP (001158374); *Strongylocentrotus *hbn (XP (781057); *Strongylocentrotus *otp (XP (784599); *Strongylocentrotus *otx (NP (999753); *Terebratalia *Pax6 (ADZ24784); *Terebratalia *homeobrain (JQ88198); *Terebratalia *otp(JQ88194); *Terebratalia *otx (ADZ24785)

#### Six-class

*Capitella *180297 (180297); *Capitella *180301 (180301); *Capitella *180303 (180303); *Capitella *226834 (226834); *Capitella *227938 (227938); *Drosophila *optix (NP_524695.2); *Drosophila *sine-oculis (NP_476733.1); *Drosophila *Six4_NP_649256.10; *Lottia *115798 (115798); *Lottia *129577 (129577); *Lottia *179424 (179424); *Mus *Six1 (NP_033215.2); *Mus *Six2 (NP_035510.1); *Mus *Six3 (NP_035511.2); *Mus *Six4 (NP_035512.1); *Mus *Six5 (NP_035513.1); *Mus *Six6 (NP_035514.1); *Nematostella *126214 (126214); *Nematostella *130873 (130873); *Nematostella *138693 (138693); *Platynereis *Six2 (CAC86663.1); *Platynereis *Six3 (CAR66435.1); *Saccoglossus *Six1 (XP_002735213.1); *Saccoglossus *Six3 (NP_001158378.1); *Saccoglossus *Six4 (XP_002735606.1); *Strongylocentrotus *Six1 (XP_001181583.1); *Strongylocentrotus *Six3 (XP_781696.1); *Strongylocentrotus *Six4 (XP_001181543.1); *Terebratalia *Six3/6 (JQ88196)

#### Synaptotagmin

*Capitella*_Dblc2 (195440); *Capitella*_Esyt2 (160561); *Capitella*_Rph (238583); *Capitella*_Syt12 (238576); *Capitella*_Syt15 (238580); *Capitella*_Syt16 (238581); *Capitella*_Syt17 (238578); *Capitella*_Syt1var1 (183139); *Capitella*_Syt1var2 (183138); *Capitella*_Syt4 (238579); *Capitella*_Syt44 (238582); *Capitella*_Syt7 (175638); *Capitella*_Syt9 (181126); *Capitella*_Sytalpha (238577); *Lottia*_Dblc2 (236949); *Lottia*_Esyt2var1 (249373); *Lottia*_Esyt2var2 (249374); *Lottia*_Rph (249375); *Lottia*_Syt12 (249365); *Lottia*_Syt15a (249370); *Lottia*_Syt15b (249367); *Lottia*_Syt16 (249369); *Lottia*_Syt17 (249364); *Lottia*_Syt18 (249372); *Lottia*_Syt1var1 (249360); *Lottia*_Syt1var2 (249359); *Lottia*_Syt21 (249371); *Lottia*_Syt4 (249363); *Lottia*_Syt47 (249366); *Lottia*_Syt7 (249362); *Lottia*_Syt9 (249361); *Lottia*_Sytalpha (249358); *Mus*_Dblc2 (NP_034199.1); *Mus*_Rph (NP_035416.1); *Mus*_Syt1 (NP_033332.1); *Mus*_Syt1 (NP_061274.2); *Mus*_Syt10 (NP_061273.1); *Mus*_Syt12 (NP_598925.1); *Mus*_Syt13 (NP_109650.1); *Mus*_Syt14 (NP_853524.1); *Mus*_Syt15 (NP_852682.1); *Mus*_Syt16 (NP_766392.2); *Mus*_Syt17 (NP_619590.1); *Mus*_Syt2 (NP_033333.2); *Mus*_Syt3 (NP_057872.2); *Mus*_Syt4 (NP_033334.2); *Mus*_Syt5 (NP_058604.1); *Mus*_Syt6 (NP_061270.2); *Mus*_Syt7 (NP_061271.1); *Mus*_Syt8 (NP_061272.2); *Mus*_Syt9 (NP_068689.2); *Terebratalia*_Syt1 (JQ88199)

## Abbreviations

BCIP: 5-bromo-4-chloro-3-indolyl phosphate; ESTs: Expressed Sequence Tags; FMRFamide: Phenylalanine-Methionine-Arginine-Phenylalanine-NH_2_; NCBI: National Center for Biotechnology Information; NBT: nitroblue tetrazolium chloride; PCR: polymerase chain reaction.

## Competing interests

The authors declare that they have no competing interests.

## Authors' contributions and declarations

SS designed the study, conducted the immunohistochemistry and microscopic imaging, contributed to the gene expression protocols, and drafted the paper. CR participated in molecular cloning, gene expression protocols, microscopic imaging, and assisted in drafting the manuscript. AH participated in molecular cloning, gene expression protocols, microscopic imaging, and assisted in drafting the manuscript. MQM assisted in drafting the manuscript. YJP conducted the molecular cloning and gene expression protocols, participated in microscopic imaging, and assisted in the drafting of the paper. All authors read and approved the final manuscript.

## Supplementary Material

Additional File 1 **Phylogenetic analysis of *Tt-hbn *and *Tt-otp***. Phylogram of *Tt-hbn, Tt-otp*, and related paired-class homeodomain proteins, supporting the orthology assignments of *Tt*-*hbn *and *Tt*-*otp*. Posterior probability for the *homeobrain *clade, including *Tt-hbn*, is 95 percent. Posterior probability for the *otp *clade, including *Tt-otp*, is 99 percent. The phylogram is a consensus of the last 2,000,000 generations from a Bayesian likelihood analysis with four independent runs of 10,000,000 generations each. Click here for file

Additional File 2 **Phylogenetic analysis of *Tt-Six3/6***. Phylogram of *Tt-Six3/6 *and related Six-class homeodomain proteins, supporting the orthology assignment of *Tt*-*Six3*/*6*. Posterior probability for the *Six3/6 *clade, including *Tt-Six3/6*, is 100 percent. The phylogram is a consensus of the last 2,000,000 generations from a Bayesian likelihood analysis with four independent runs of 10,000,000 generations each. Click here for file

Additional File 3 **Phylogenetic analysis of *Tt-fez***. Phylogram of *Tt-fez *and related zinc-finger proteins, supporting the orthology assignment of *Tt*-*fez*. Posterior probability for the Fez clade, including *Tt-f*ez, is 100 percent. The phylogram is a consensus of the last 2,000,000 generations from a Bayesian likelihood analysis with four independent runs of 10,000,000 generations each. Click here for file

Additional File 4 **Phylogenetic analysis of *Tt-NK2.1***. Phylogram of *Tt-NK2.1 *and related *NK*-class homeodomain proteins, supporting the orthology assignment of *Tt*-*NK2.1*. Posterior probability for the *NK2.1 *clade, including *Tt-NK2.1*, is 70 percent. The phylogram is a consensus of the last 2,000,000 generations from a Bayesian likelihood analysis with four independent runs of 10,000,000 generations each. Click here for file

Additional File 5 **Phylogenetic analysis of *Tt-FoxG *and *Tt-FoxQ2***. Phylogram of *Tt*-FoxG, *Tt-FoxQ2*, and related Forkead box proteins, supporting the orthology assignments of *Tt*-*FoxG *and *Tt*-*FoxQ2*. Posterior probability for the *FoxG *clade, including *Tt-FoxG*, is 100 percent. Posterior probability for the *FoxQ2 *clade, including *Tt-FoxQ2*, is 94 percent. The phylogram is a consensus of the last 2,000,000 generations from a Bayesian likelihood analysis with four independent runs of 20,000,000 generations each. Click here for file

Additional File 6 **Phylogenetic analysis of *Tt-synaptotagmin 1***. Phylogram of *Tt-synaptotagmin 1 *and related synaptotagmin proteins, supporting the orthology assignment of *Tt*-*synaptotagmin 1*. Posterior probability for the *synaptotagmin 1 *clade, including *Tt-synaptotagmin 1*, is 100 percent (inclusive of the presumptive paralogs *Mus Syt2, Mus Syt5 *and *Mus Syt8*). The phylogram is a consensus of the last 2,000,000 generations from a Bayesian likelihood analysis with four independent runs of 10,000,000 generations each. Click here for file
