# Supplementary material for: Development of the larval anterior neurogenic domains of Terebratalia transversa (Brachiopoda) provides insights into the diversification of larval apical organs and the spiralian nervous system
Source: EvoDevo. 2012 Jan 24;3:3. doi: 10.1186/2041-9139-3-3 (PMC3314550; doi:10.1186/2041-9139-3-3)
Supplement: Additional File 1 — Phylogenetic analysis of Tt-hbn and Tt-otp. Phylogram of Tt-hbn, Tt-otp, and related paired-class homeodomain proteins, supporting the orthology assignments of Tt-hbn and Tt-otp. Posterior probability for the homeobrain clade, including Tt-hbn, is 95 percent. Posterior probability for the otp clade, including Tt-otp, is 99 percent. The phylogram is a consensus of the last 2,000,000 generations from a Bayesian likelihood analysis with four independent runs of 10,000,000 generations each. [file 2041-9139-3-3-S1.PDF]

Paired class genes: *homeobrain* and *otp*

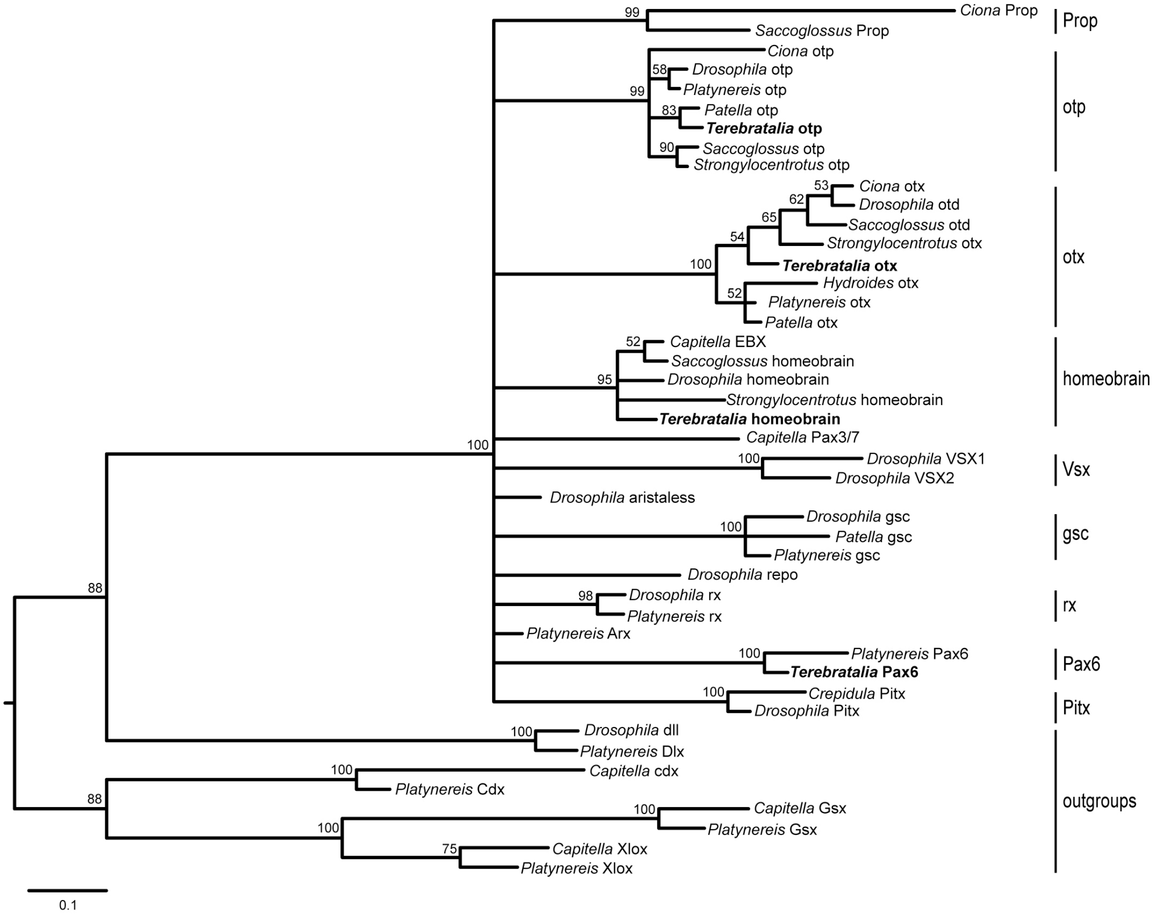

Additional File 1: Phylogenetic analysis of *Tt-hbn* and *Tt-otp*. Phylogram of *Tt-hbn*, *Tt-otp*, and related paired-class homeodomain proteins, supporting the orthology assignments of *Tt-hbn* and *Tt-otp*. Posterior probability for the *homeobrain* clade, including *Tt-hbn*, is 95 percent. Posterior probability for the *otp* clade, including *Tt-otp*, is 99 percent. The phylogram is a consensus of the last 2,000,000 generations from a Bayesian likelihood analysis with four independent runs of 10,000,000 generations each.
