# Supplementary material for: Development of the larval anterior neurogenic domains of Terebratalia transversa (Brachiopoda) provides insights into the diversification of larval apical organs and the spiralian nervous system
Source: EvoDevo. 2012 Jan 24;3:3. doi: 10.1186/2041-9139-3-3 (PMC3314550; doi:10.1186/2041-9139-3-3)
Supplement: Additional File 2 — Phylogenetic analysis of Tt-Six3/6. Phylogram of Tt-Six3/6 and related Six-class homeodomain proteins, supporting the orthology assignment of Tt-Six3/6. Posterior probability for the Six3/6 clade, including Tt-Six3/6, is 100 percent. The phylogram is a consensus of the last 2,000,000 generations from a Bayesian likelihood analysis with four independent runs of 10,000,000 generations each. [file 2041-9139-3-3-S2.PDF]

Six class genes: *Six 3/6*

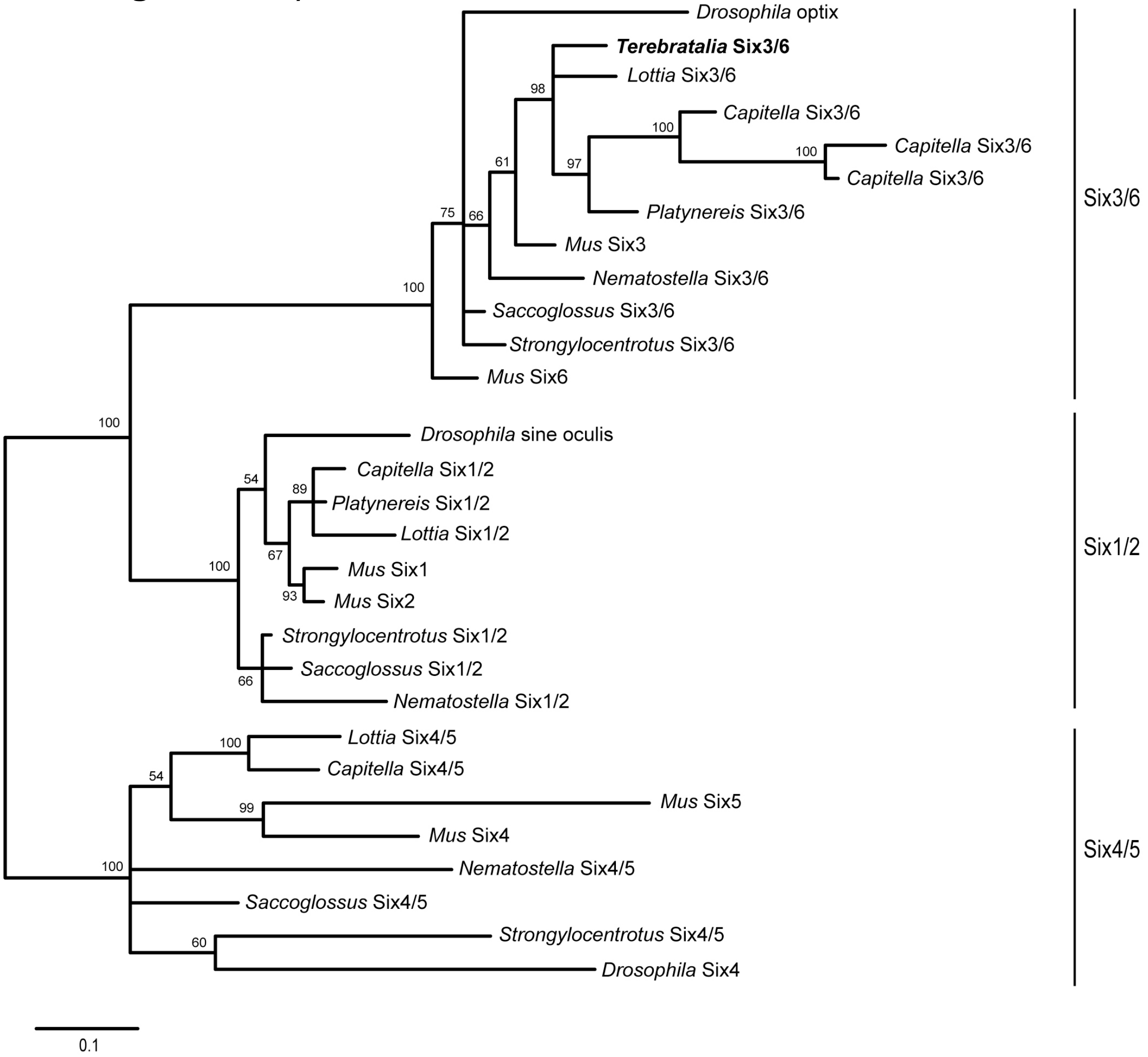

Additional File 2: Phylogenetic analysis of *Tt-Six3/6*.

Phylogram of *Tt-Six3/6* and related Six-class homeodomain proteins, supporting the orthology assignment of *Tt-Six3/6*. Posterior probability for the *Six3/6* clade, including *Tt-Six3/6*, is 100 percent. The phylogram is a consensus of the last 2,000,000 generations from a Bayesian likelihood analysis with four independent runs of 10,000,000 generations each.
