# Supplementary material for: Development of the larval anterior neurogenic domains of Terebratalia transversa (Brachiopoda) provides insights into the diversification of larval apical organs and the spiralian nervous system
Source: EvoDevo. 2012 Jan 24;3:3. doi: 10.1186/2041-9139-3-3 (PMC3314550; doi:10.1186/2041-9139-3-3)
Supplement: Additional File 3 — Phylogenetic analysis of Tt-fez. Phylogram of Tt-fez and related zinc-finger proteins, supporting the orthology assignment of Tt-fez. Posterior probability for the Fez clade, including Tt-fez, is 100 percent. The phylogram is a consensus of the last 2,000,000 generations from a Bayesian likelihood analysis with four independent runs of 10,000,000 generations each. [file 2041-9139-3-3-S3.PDF]

Fez

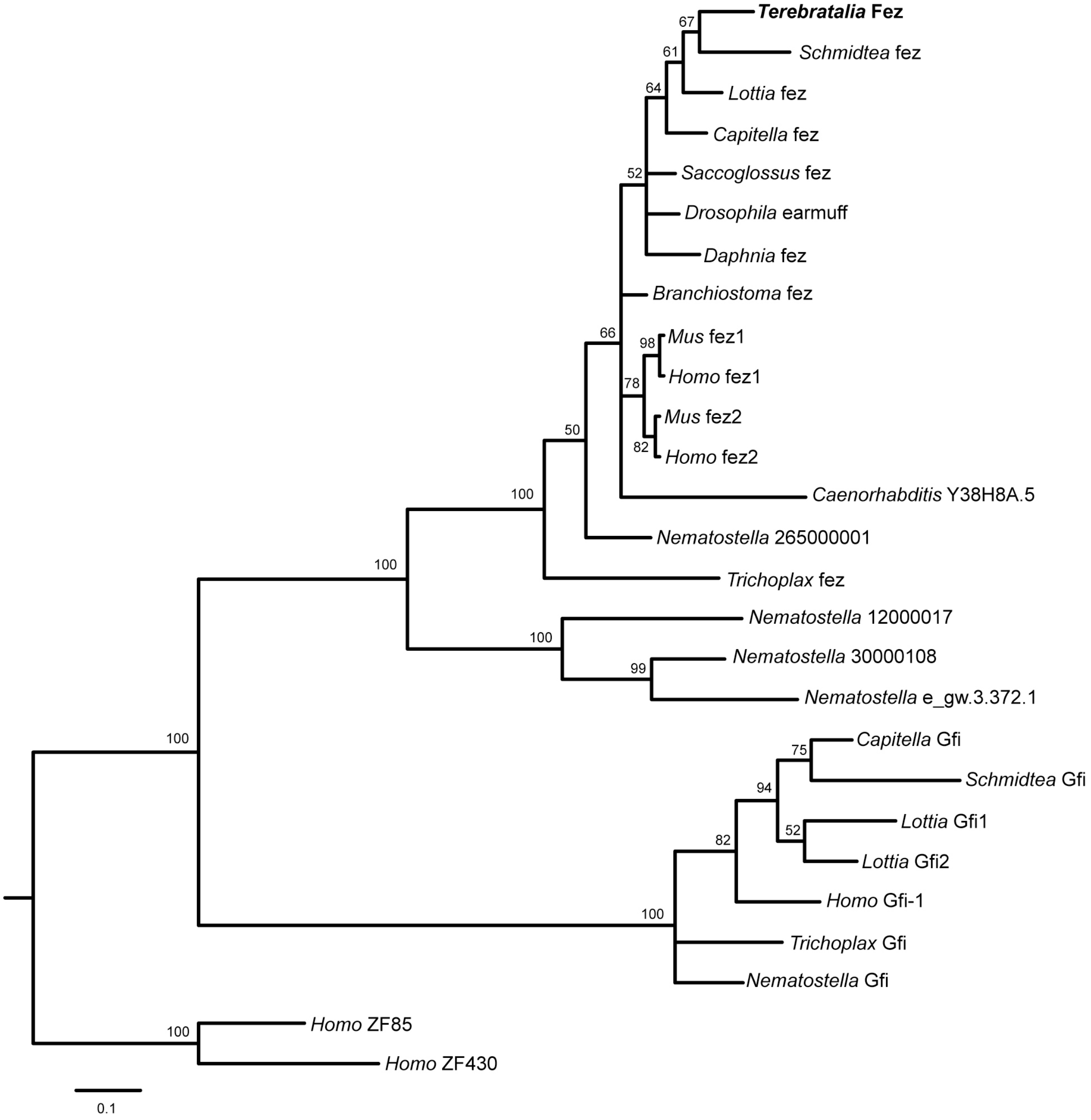

Additional File 3: Phylogenetic analysis of *Tt-fez*.

Phylogram of *Tt-fez* and related zinc-finger proteins, supporting the orthology assignment of *Tt-fez*. Posterior probability for the Fez clade, including *Tt-f*ez, is 100 percent. The phylogram is a consensus of the last 2,000,000 generations from a Bayesian likelihood analysis with four independent runs of 10,000,000 generations each.
