# Supplementary material for: Development of the larval anterior neurogenic domains of Terebratalia transversa (Brachiopoda) provides insights into the diversification of larval apical organs and the spiralian nervous system
Source: EvoDevo. 2012 Jan 24;3:3. doi: 10.1186/2041-9139-3-3 (PMC3314550; doi:10.1186/2041-9139-3-3)
Supplement: Additional File 4 — Phylogenetic analysis of Tt-NK2.1. Phylogram of Tt-NK2.1 and related NK-class homeodomain proteins, supporting the orthology assignment of Tt-NK2.1. Posterior probability for the NK2.1 clade, including Tt-NK2.1, is 70 percent. The phylogram is a consensus of the last 2,000,000 generations from a Bayesian likelihood analysis with four independent runs of 10,000,000 generations each. [file 2041-9139-3-3-S4.PDF]

**NK class: *NK2.1***

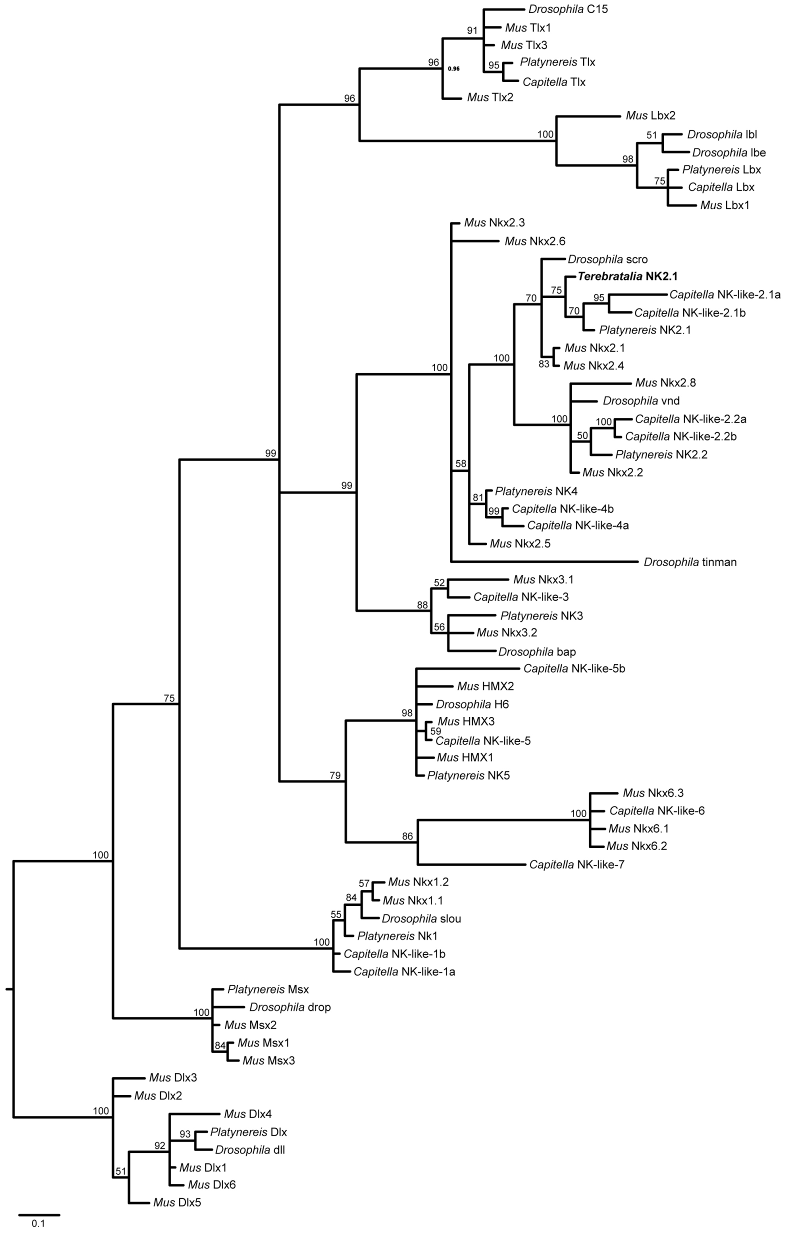


**Additional File 4: Phylogenetic analysis of *Tt-NK2.1*.**

Phylogram of *Tt-NK2.1* and related *NK*-class homeodomain proteins, supporting the orthology assignment of *Tt-NK2.1*. Posterior probability for the *NK2.1* clade, including *Tt-NK2.1*, is 70 percent. The phylogram is a consensus of the last 2,000,000 generations from a Bayesian likelihood analysis with four independent runs of 10,000,000 generations each.
