# Supplementary material for: Development of the larval anterior neurogenic domains of Terebratalia transversa (Brachiopoda) provides insights into the diversification of larval apical organs and the spiralian nervous system
Source: EvoDevo. 2012 Jan 24;3:3. doi: 10.1186/2041-9139-3-3 (PMC3314550; doi:10.1186/2041-9139-3-3)
Supplement: Additional File 5 — Phylogenetic analysis of Tt-FoxG and Tt-FoxQ2. Phylogram of Tt-FoxG, Tt-FoxQ2, and related Forkead box proteins, supporting the orthology assignments of Tt-FoxG and Tt-FoxQ2. Posterior probability for the FoxG clade, including Tt-FoxG, is 100 percent. Posterior probability for the FoxQ2 clade, including Tt-FoxQ2, is 94 percent. The phylogram is a consensus of the last 2,000,000 generations from a Bayesian likelihood analysis with four independent runs of 20,000,000 generations each. [file 2041-9139-3-3-S5.PDF]

Fox Class genes: *FoxQ2* and *FoxG*

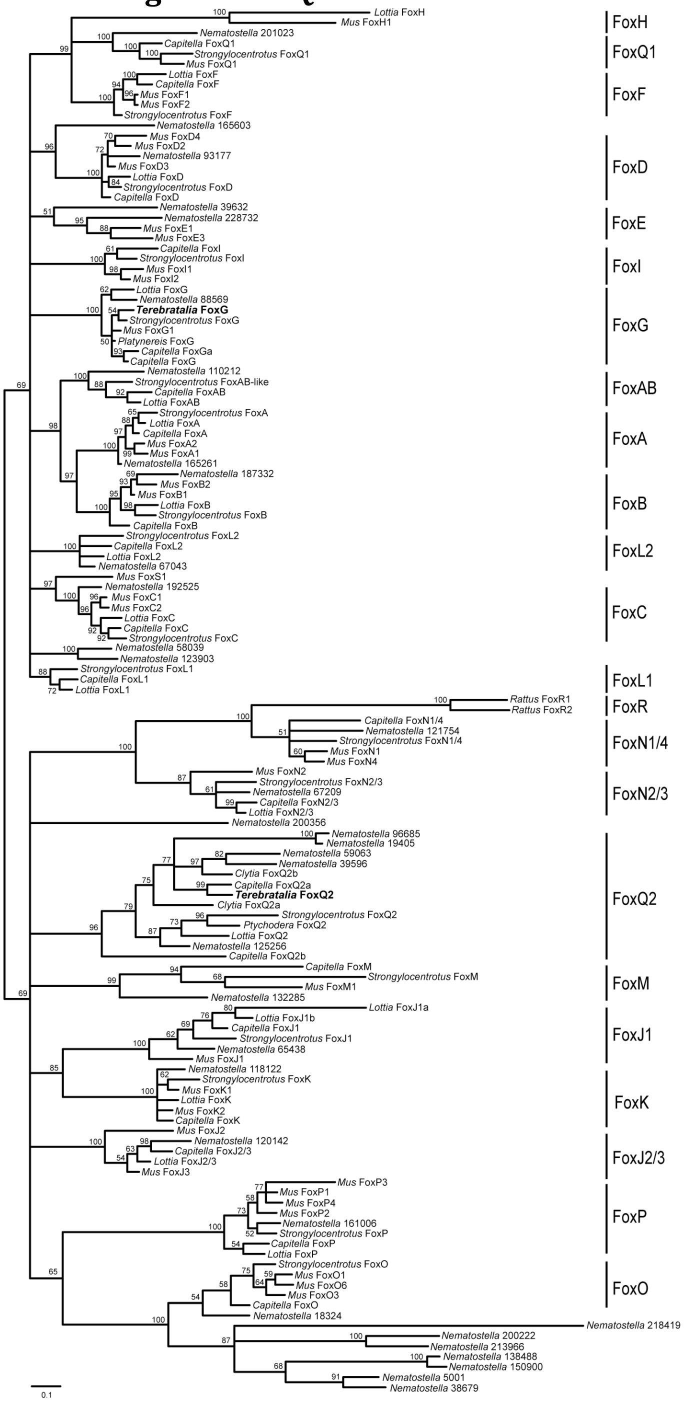

Additional File 5: Phylogenetic analysis of *Tt-FoxG* and *Tt-FoxQ2*.
Phylogram of *Tt*-FoxG, *Tt-FoxQ2*, and related Forkead box proteins, supporting the orthology assignments of *Tt-FoxG* and *Tt-FoxQ2*. Posterior probability for the *FoxG* clade, including *Tt-FoxG*, is 100 percent. Posterior probability for the *FoxQ2* clade, including *Tt-FoxQ2*, is 94 percent. The phylogram is a consensus of the last 2,000,000 generations from a Bayesian likelihood analysis with four independent runs of 20,000,000 generations each.
