# Supplementary material for: Development of the larval anterior neurogenic domains of Terebratalia transversa (Brachiopoda) provides insights into the diversification of larval apical organs and the spiralian nervous system
Source: EvoDevo. 2012 Jan 24;3:3. doi: 10.1186/2041-9139-3-3 (PMC3314550; doi:10.1186/2041-9139-3-3)
Supplement: Additional File 6 — Phylogenetic analysis of Tt-synaptotagmin 1. Phylogram of Tt-synaptotagmin 1 and related synaptotagmin proteins, supporting the orthology assignment of Tt-synaptotagmin 1. Posterior probability for the synaptotagmin 1 clade, including Tt-synaptotagmin 1, is 100 percent (inclusive of the presumptive paralogs Mus Syt2, Mus Syt5 and Mus Syt8). The phylogram is a consensus of the last 2,000,000 generations from a Bayesian likelihood analysis with four independent runs of 10,000,000 generations each. [file 2041-9139-3-3-S6.PDF]

Synaptotagmin

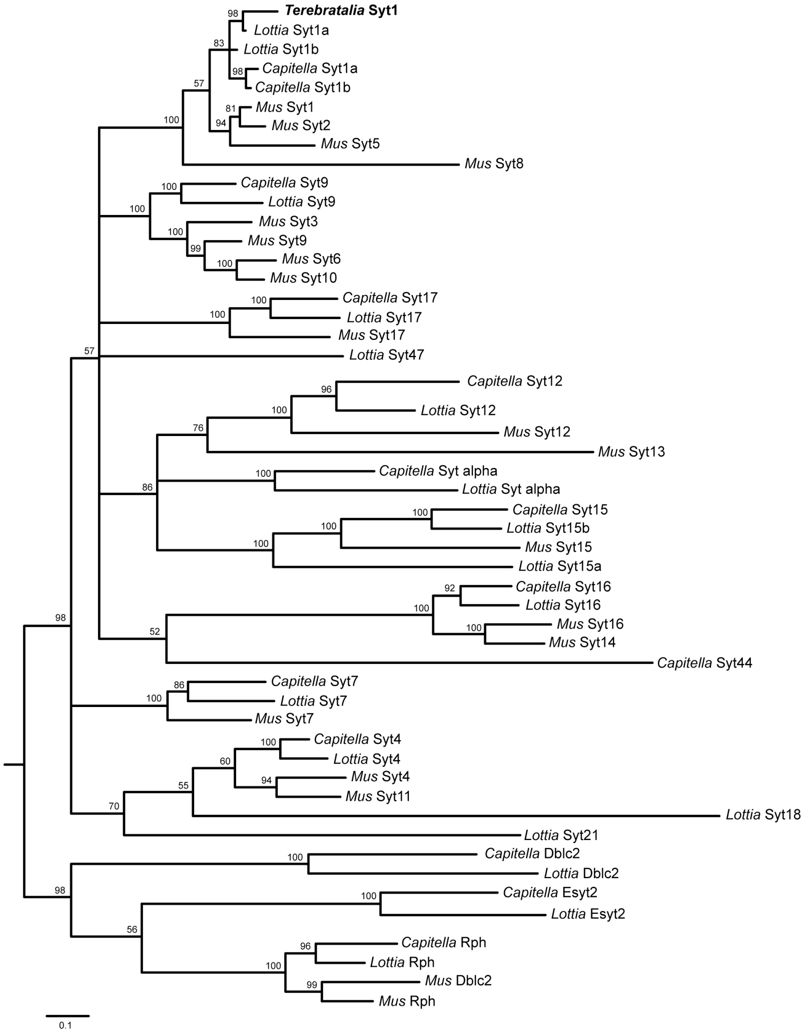

Additional File 6: Phylogenetic analysis of *Tt-synaptotagmin 1*.

Phylogram of *Tt-synaptotagmin 1* and related synaptotagmin proteins, supporting the orthology assignment of *Tt-synaptotagmin 1*. Posterior probability for the *synaptotagmin 1* clade, including *Tt-synaptotagmin 1*, is 100 percent (inclusive of the presumptive paralogs *Mus Syt2*, *Mus Syt5* and *Mus Syt8*). The phylogram is a consensus of the last 2,000,000 generations from a Bayesian likelihood analysis with four independent runs of 10,000,000 generations each.
